# Promoting Graduate Student Mental Health During COVID-19: Acceptability, Feasibility, and Perceived Utility of an Online Single-Session Intervention

**DOI:** 10.3389/fpsyg.2021.569785

**Published:** 2021-04-07

**Authors:** Akash R. Wasil, Madison E. Taylor, Rose E. Franzen, Joshua S. Steinberg, Robert J. DeRubeis

**Affiliations:** ^1^Department of Psychology, University of Pennsylvania, Philadelphia, PA, United States; ^2^Children’s Hospital of Philadelphia, Philadelphia, PA, United States

**Keywords:** public health, digital mental health, evidence-based practices, COVID-19, graduate students, common elements, cognitive-behavioral therapy, positive psychology

## Abstract

The COVID-19 outbreak has simultaneously increased the need for mental health services and decreased their availability. Brief online self-help interventions that can be completed in a single session could be especially helpful in improving access to care during the crisis. However, little is known about the uptake, acceptability, and perceived utility of these interventions outside of clinical trials in which participants are compensated. Here, we describe the development, deployment, acceptability ratings, and pre–post effects of a single-session intervention, the Common Elements Toolbox (COMET), adapted for the COVID-19 crisis to support graduate and professional students. Participants (*n* = 263), who were not compensated, were randomly assigned to two of three modules: behavioral activation, cognitive restructuring, and gratitude. Over 1 week, 263 individuals began and 189 individuals (72%) completed the intervention. Participants reported that the intervention modules were acceptable (93% endorsing), helpful (88%), engaging (86%), applicable to their lives (87%), and could help them manage COVID-related challenges (88%). Participants reported pre- to post-program improvements in secondary control (i.e., the belief that one can control their reactions to objective events; *d*_*av*_ = 0.36, *d*_*z*_ = 0.50, *p* < 0.001) and in the perceived negative impact of the COVID-19 crisis on their quality of life (*d*_*av*_ = 0.22, *d*_*z*_ = 0.25, *p* < 0.001). On average, differences in their perceived ability to handle lifestyle changes resulting from the pandemic were positive, but small and at the level of a non-significant trend (*d*_*av*_ = 0.13, *d*_*z*_ = 0.14, *p* = 0.066). Our results highlight the acceptability and utility of an online intervention for supporting individuals through the COVID-19 crisis.

## Introduction

In addition to the serious physical health consequences of COVID-19, the resulting societal changes have had major impacts on population-wide mental health (Liu et al., 2020). COVID-19 has introduced a variety of stressors into modern life, including fears about contracting the virus, concern for loved ones, economic instability, social distancing, and other major lifestyle disruptions (Pfefferbaum and North, 2020). Many of these concerns have affected graduate and professional students (i.e., students earning advanced degrees, as well as non-traditional and non-degree seeking students). Even before the crisis, students were vulnerable to depression, anxiety, loneliness, and suicidal ideation (Evans et al., 2018). The COVID-19 crisis has exacerbated these concerns: many universities have ceased non-essential operations, mandated that students leave campus, and shut down university counseling centers (Zhai and Du, 2020). These measures, while appropriate to avoid the spread of COVID-19, have led to considerable uncertainty, stress, and disruption for graduate and professional students (Chirikov et al., 2020; Sahu, 2020). In open-ended surveys, graduate and professional students report a variety of concerns relating to their health, productivity, and well-being (Wasil et al., 2021a). Furthermore, in a recent survey of over 15,000 graduate students, about 32% of students reported elevated depressive symptoms and 39% reported elevated anxiety symptoms (Chirikov et al., 2020). The estimated rates of depression and anxiety in 2020 were 1.5–2 times higher in 2020 than in 2019 (Chirikov et al., 2020). To support graduate and professional students, evidence-based mental health and wellness promotion efforts are needed.

Traditional mental health services, while essential, will not be sufficient to meet the growing need for mental health care during this crisis. As a result of social distancing requirements, many clinicians and mental health care organizations have moved counseling activities to telehealth platforms (Torous et al., 2020). However, even before the COVID-19 crisis, the demand for mental health services greatly exceeded the availability of professionals, with only a small fraction of individuals in need of mental health services receiving care (Kazdin and Blase, 2011). Due to the pre-existing lack of mental health care providers, the growing demand for services, and the strain on the health care system, telehealth platforms cannot serve as the only option during the pandemic. As such, scalable methods of delivering mental health interventions could be especially impactful during the COVID-19 crisis.

One promising approach involves the development and dissemination of evidence-based digital self-help interventions. Digital self-help interventions have several features that make them appealing in times of crisis. First, these interventions do not require in-person support and thus do not place additional burden on the health care system. Second, these interventions can be disseminated cheaply (or for free) to wide audiences. Third, digital interventions can be updated and adapted in short periods of time. This could be especially useful during the pandemic, as evidence-based content could be adapted specifically to address concerns relating to COVID-19. Finally, digital interventions are effective for a variety of mental health conditions, including depression and anxiety (Josephine et al., 2017; Karyotaki et al., 2017), and some have been shown to be effective in just a single session (Schleider and Weisz, 2018; Osborn et al., 2020). Thus, digital interventions could provide low-intensity evidence-based care to individuals who need support during the crisis but lack access to other services.

However, the uptake of digital interventions in real-world settings has been poor. Although evidence from clinical trials supports the acceptability of digital mental health interventions, findings from controlled clinical trials may not generalize to use in naturalistic settings. Recent evidence shows that uptake and engagement rates for digital interventions are lower in open field studies than in formal clinical trials (Fleming et al., 2018). Because participants in clinical trials are often financially compensated for consistent participation and clinical trials attract highly motivated participants, formal trials yield unrealistically high estimates of engagement. In order to improve the dissemination of digital interventions, studies focused on the acceptability and uptake of digital interventions in real-world contexts are needed (Mohr et al., 2017). Although there are some digital interventions with relatively high uptake (as measured by number of downloads), these interventions have not been rigorously evaluated, contain a limited amount of evidence-based content (Wasil et al., 2019), and generally fail to retain users (Baumel et al., 2019; Wasil et al., 2020a, 2021b). Indeed, most mental health apps have only 4% of users continue to engage with the apps after 15 days, and many have no active monthly users (Baumel et al., 2019; Wasil et al., 2020b, 2021c). Some barriers that limit engagement for digital interventions include lack of perceived utility, limits on accessibility (e.g., fees or geographic restrictions), poor user experiences, and high demands on users’ time (Torous et al., 2018). As a result, there is a need for digital mental health interventions that (a) include evidence-based content, (b) demonstrate acceptability and feasibility outside of formal clinical trials, (c) minimize demands on users’ time, and (d) are available at no cost.

Single-session interventions, which can be completed in one sitting, may be particularly useful. Meta-analytic evidence suggests that single-session interventions have effects similar in magnitude to those of psychotherapies that last several months (see Schleider and Weisz, 2017), and some online single-session interventions have shown effects on depression even at 4–9 months follow-ups (e.g., Schleider and Weisz, 2018). In addition to their effectiveness, single-session interventions are highly scalable, can be disseminated at very low costs, and can be adapted readily for new populations (see Osborn et al., 2020; Wasil et al., 2020c). Online single-session interventions also circumvent concerns about user retention, as users only need to engage with the intervention once to receive the full dose of content. However, there are several gaps in research on online single-session interventions. For instance, limited research has examined the acceptability of online single-session interventions outside the context of controlled clinical trials. Additionally, while online single-session interventions often include content that has been shown to be effective for individuals across age groups, most research on online single-session interventions has been conducted with youth and adolescents (Schleider and Weisz, 2017). Finally, few online single-session interventions have been adapted to respond to a public health emergency, such as the COVID-19 crisis. As such, a digital single-session intervention adapted specifically to meet the needs of the current crisis would provide a considerable contribution to the field.

Such an intervention would be especially valuable if it included modules that are commonly included in empirically supported interventions. Whereas mental health interventions have traditionally been tested in multicomponent packages, often in the form of published treatment manuals, scholars have recently pushed for a focus on specific procedures within interventions (Hofmann and Hayes, 2019). Relatedly, some scholars have identified treatment procedures that are commonly included in treatment manuals of empirically supported interventions (Chorpita and Daleiden, 2009). These procedures, known as “common elements,” have formed the basis of recently-developed modular interventions. Such interventions include the *Modular Approach to Therapy for Children with Anxiety, Depression, Trauma, or Conduct Problems* (MATCH; Weisz et al., 2012) and the *Common Elements Treatment Approach* (CETA; Murray et al., 2014). Advantages of modular interventions include their flexibility, scalability, and adaptability (see Weisz et al., 2012). Modular interventions could be especially useful in the context of the COVID-19 crisis, as modules can be adapted or replaced to meet the needs of specific populations (e.g., patients, healthcare workers, individuals under stay-at-home orders). While previous research has shown that modular interventions are effective when delivered by clinicians (Weisz et al., 2012) and trained lay counselors (Murray et al., 2014), relatively little is known about modular interventions as self-administered digital interventions.

To fill these gaps, and to provide timely support to students dealing with the COVID-19 crisis, our team adapted and deployed an online single-session intervention for graduate and professional students.

We believed that a self-administered intervention focused on common elements could be valuable. In this paper, we describe the *Common Elements Toolbox* (COMET), a self-administered digital single-session intervention. Here, we describe (a) the development and design of COMET, (b) adaptations we made to COMET to respond to the COVID-19 crisis, (c) an evaluation of the acceptability and perceived utility of COMET, and (d) changes in secondary control and COVID-19 related concerns from pre-intervention to post-intervention. In addition to filling important gaps in the literature, our goal is to provide information that can directly inform other digital mental health promotion efforts in response to the COVID-19 pandemic.

## Materials and Methods

### Selection of COMET Intervention Modules

In September of 2019, our team began to develop an online single-session intervention to promote college student mental health. To begin this process, we reviewed literatures on common elements in empirically supported treatments (Chorpita and Daleiden, 2009; Higa-McMillan et al., 2016), treatment elements in existing digital interventions (Wasil et al., 2019), psychologically “wise” single-component interventions (Walton and Wilson, 2018), positive psychology interventions (Seligman et al., 2005), and single-session interventions (Schleider and Weisz, 2017). As much of this literature focused on youth and adolescent populations, we supplemented our review by examining treatment manuals for adults (Nathan and Gorman, 2015). Our aim in this review was to identify intervention techniques to include in the single-session intervention that were shown to be effective, ideally when administered on their own, and could be taught easily in a self-guided format.

With these criteria in mind, we chose three modules to include in COMET: behavioral activation, cognitive restructuring, and gratitude. Behavioral activation and cognitive restructuring are both considered core components of cognitive-behavioral therapies. Numerous studies have demonstrated the effectiveness of web-based cognitive behavioral therapy in treating depression and anxiety (see Karyotaki et al., 2017 for a meta-analysis). Gratitude is a common element in several empirically supported positive psychology interventions (see Supplementary Materials for details). Additionally, meta-analytic evidence suggests that gratitude interventions reduce symptoms of depression and anxiety (Cregg and Cheavens, 2020) and improve subjective well-being (Davis et al., 2016; Dickens, 2017).

From September 2019 to February 2020, we prepared an intervention with these three modules for a pre-registered randomized controlled trial with undergraduate students (NCT04287374; see Supplementary Materials for details). The undergraduate version of COMET was based on previous CBT and gratitude interventions, and the design of the intervention was informed by that of previous web-based single-session interventions (Seligman et al., 2005; Nathan and Gorman, 2015; Schleider and Weisz, 2017, 2018; Osborn et al., 2020; Schleider et al., 2020; Wasil et al., 2020d). As we finished developing the undergraduate version of COMET in March 2020, COVID-19 began to rapidly spread throughout the United States, including the greater Philadelphia area. In light of the mental health crisis due to COVID-19 disruptions, we decided to adapt the existing intervention into a separate intervention for graduate and professional students.

Over the course of 2 weeks, adaptations were made to COMET to increase its relevance to the COVID-19 crisis. For example, the initial intervention included a vignette about a student struggling with rejection from summer internships. For the adapted version, we replaced this vignette with one about a student who is struggling to adjust to lifestyle changes resulting from the COVID-19 crisis. We also consulted relevant stakeholders: we received feedback from undergraduate students, graduate students, and faculty in order to refine the intervention. In response to input from university deans about the length of the intervention, we decided to provide only two of the three modules to each participant. This was done to maximize the likelihood that students would choose to participate, thus increasing the reach of our intervention. The specific modules provided to a given student were determined randomly (see Supplementary Materials for details).

### Adapted Version of COMET for COVID-19

At the beginning of the adapted intervention, participants read a short introduction message describing the purpose of the intervention. Participants were then randomly assigned to receive two of the three intervention modules: behavioral activation, cognitive restructuring, and gratitude.

#### Behavioral Activation

The purpose of the behavioral activation module (labeled “positive activities” to sound less technical and to improve comprehension) was to help participants identify and perform activities that bring them happiness or provide a sense of mastery. Participants received psychoeducation about pleasurable activities (i.e., enjoyable activities) and mastery activities (i.e., activities that provide a sense of accomplishment). Next, they were prompted to brainstorm and list positive activities, with at least one being a pleasurable activity and at least one being a mastery activity. Participants were then instructed to select an activity to perform more frequently in the upcoming weeks. Then, they reflected on why this activity is important to them and how this activity influences their mood. Finally, participants completed a plan describing when they will perform the activity, where they will be, who they could tell about their plan to help them stay accountable, and how they will overcome obstacles that might get in the way of their plan (see Supplementary Figure 1).

#### Cognitive Restructuring

The purpose of the cognitive restructuring module (labeled “flexible thinking” to sound less technical and to improve comprehension) was to help participants notice and reframe unrealistic negative thoughts. Participants first received brief psychoeducation about negative thoughts and thinking traps. They were then presented with a short vignette about “Gwen,” a hypothetical graduate student whose routine has been affected by coronavirus. In the vignette, participants read that Gwen feels like she has lost control over her routine and feels frustrated with herself for not keeping up with her work and health goals while under quarantine. Gwen also expresses worries that her friendships may suffer while she’s in isolation (see Supplementary Figure 2).

After reading the vignette, participants learned a cognitive restructuring technique called the “ABCD Technique” and applied it to Gwen’s situation. They were prompted to identify details about the Activating Event (i.e., list objective facts about Gwen’s situation), list Beliefs (i.e., initial worries or concerns that Gwen might have), generate ways to Challenge the negative or unrealistic beliefs (i.e., ways Gwen could reframe her initial concerns), and Debrief (i.e., indicate how Gwen might be feeling as a result of challenging her beliefs). After each step, participants viewed an example of a completed response (e.g., after they listed ways Gwen could challenge her beliefs, they were provided with examples of ways that Gwen could challenge her beliefs). These examples were presented to participants to provide them with real-time feedback and a model response (see Supplementary Figure 3). After applying the ABCD Technique to Gwen’s vignette, participants were prompted to use the ABCD Technique to restructure their thoughts about a distressing situation in their own lives.

#### Gratitude

The purpose of the gratitude module was to encourage participants to notice and appreciate positive things in their lives. Participants started the module by briefly reading about benefits of gratitude and past research on gratitude interventions. Then, participants completed the Three Good Things exercise (see Seligman et al., 2005). They received instructions to identify three good things that happened to them in the past 2 days. For each good thing, they also wrote a brief reflection explaining why it was meaningful to them and how to increase the odds of it happening again. Before writing their good things, participants viewed an example written by a hypothetical student (see Supplementary Figure 4). Finally, participants completed a novel *present-focused gratitude* exercise. In this exercise, participants were prompted to take a few moments to notice and appreciate things around them (e.g., “I love the color of that chair,” “I’m very warm and comfortable right now”). Because the present-focused gratitude exercise involves noticing things in one’s immediate environment, we believed that it would be especially relevant during the COVID-19 crisis, in which many individuals are staying in the same environment (e.g., at home).

### Recruitment

We partnered with the Behavior Change for Good Initiative and administrators at the University of Pennsylvania to email students in the Graduate School of Arts and Sciences and the College of Liberal and Professional Studies. The email, sent via official student listservs at the end of March 2020, invited students to take advantage of “an online tool grounded in behavioral science” and mentioned that “this is an option, not an obligation.” The email also included a link to the intervention, hosted on Qualtrics. Based on estimates provided by the university, we expect that approximately 3,000 students received the invitation. To maximize the reach of the intervention, all graduate and professional students were eligible to participate; there were no exclusion criteria. In the present study, we analyze responses from the first week of recruitment (i.e., March 30 to April 6).

### Procedure

Upon opening the Qualtrics link, participants were directed to a brief introductory screen with information about the study’s purpose and a general description of the activities. Participants then filled out a brief baseline questionnaire with measures of depressive symptoms, anxiety symptoms, secondary control, and perceived ability to handle the COVID-19 crisis (described in further detail below). Once the baseline questionnaire was completed, participants were randomized to receive two of the three intervention modules described above. Module order was also randomized, resulting in six possible combinations of the three modules (i.e., 1/6 of participants received cognitive restructuring followed by gratitude while 1/6 received gratitude followed by cognitive restructuring, etc.). After each intervention module, participants were asked to rate the module on acceptability, perceived helpfulness, engagement, and applicability. After completing both modules, participants filled out a brief post-intervention questionnaire, including some of the pre-intervention measures, feedback questions about the intervention, and demographic information (described in detail below). Upon finishing the intervention, participants received an automated email encouraging them to continue practicing the activities they performed; the email included worksheets to help them practice. Because our primary intention was to provide support to students in light of the pandemic, we chose not to include a control group or require participants to complete follow-up assessments. With this in mind, we applied for Quality Assurance/Quality Improvement approval from the Institutional Review Board (see Supplementary Materials for details). Study procedures were reviewed and deemed quality improvement by the University of Pennsylvania Institutional Review Board.

### Measures

#### Baseline Measures

##### Depressive symptoms (patient health questionnaire-2)

The Patient Health Questionnaire-2 (PHQ-2; Kroenke et al., 2003), a commonly used measure of depression, was administered to participants at baseline. The PHQ-2 asks participants to report the frequency of depressed mood and anhedonia over the past 2 weeks. Each item is scored from 0 (“not at all”) to 3 (“nearly every day”). The PHQ-2 has demonstrated strong psychometric properties, including construct validity. PHQ-2 scores are associated with functional impairment, symptom-related difficulties, and clinician ratings of depression (Kroenke et al., 2003). Cronbach’s alpha in our sample was 0.8.

##### Anxiety symptoms (generalized anxiety disorder-2)

The Generalized Anxiety Disorder 2-item scale (GAD-2; Kroenke et al., 2007), a commonly used measure of anxiety, was administered to participants at baseline. The GAD-2 asks participants to report the frequency of anxiety and inability to stop worrying over the past 2 weeks. Each item is scored from 0 (“not at all”) to 3 (“nearly every day”). The GAD-2 has demonstrated strong psychometric properties, including construct validity. GAD-2 scores are associated with functional impairment, and clinician ratings of anxiety (Plummer et al., 2016). Cronbach’s alpha in our sample was 0.87.

#### Post-module Measures

##### Acceptability and perceived utility

After each module, participants were asked to complete the Acceptability of Intervention Measure (AIM; Weiner et al., 2017). The items of the AIM prompt participants to rate the degree to which they approved of, welcomed, liked, and found a module appealing. Participants respond to the four items on a 5-point Likert scale, ranging from 1 (completely disagree) to 5 (completely agree). The four ratings are averaged to yield an acceptability score (Weiner et al., 2017).

We also administered three items to assess the perceived helpfulness, engagement, and applicability of each intervention module. Item wording was adjusted depending on the modules that participants received. For instance, participants who received the behavioral activation module were asked to rate the following statements: “I found the positive activities exercise helpful,” (perceived helpfulness), “I found the positive activities exercise engaging,” (engagement), and “I think I will continue applying content from the positive activities exercise in the weeks ahead” (applicability). Each item was rated on a 7-point Likert scale ranging from 1 (“strongly disagree”) to 7 (“strongly agree.”).

#### Pre- and Post-intervention Measures

##### Secondary control (secondary control scale)

Secondary control refers to individuals’ perceived ability to manage the personal or psychological impact from objective conditions or events (Weisz et al., 2010). Secondary control has been contrasted with primary control, the ability of individuals to influence objective conditions or events in their lives (Rothbaum et al., 1982). Secondary control may be especially important during times of crisis, when individuals have relatively less primary control over their objective situations. For example, individuals during the COVID-19 crisis do not have much control over where they can travel or whom they can visit (primary control). However, they are able to control how they respond to the pandemic and the lifestyle changes caused by social distancing (secondary control).

To assess secondary control, we adapted three items from the Secondary Control Scale for Children (SCSC; Weisz et al., 2010), which we administered at baseline and post-intervention. The SCSC asks participants to rate the extent to which various statements are true about how they react to negative events. Each item is scored on a 4-point Likert scale from 0 (“very false”) to 3 (“very true”). Although the scale was initially designed for youth and adolescents, it contains items that are relevant across age groups. To reduce participant burden and to ensure that the questions were relevant to our sample of graduate and professional students, we selected three items from the SCSC (see Supplementary Materials). The SCSC has demonstrated strong psychometric properties in samples of youth and adolescents (Weisz et al., 2010; Schleider and Weisz, 2018). Cronbach’s alpha for the three items in our sample was 0.74.

##### Ability to handle COVID-19 and perceived impact

We developed and administered three questions relating to the COVID-19 pandemic. The first two questions were administered at baseline and post-intervention. The first question asked participants about their perceived ability to handle lifestyle changes that result from the COVID-19 crisis (“Over the next 2 weeks, I think I will be able to handle lifestyle changes that have resulted from the coronavirus pandemic”). The second asked participants to predict the impact of the coronavirus on their overall quality of life (“Over the next 2 weeks, I think the pandemic will have an extremely negative impact on my quality of life”). Both statements were rated on a 7-point Likert scale ranging from 1 (“strongly disagree”) to 7 (“strongly agree”).

#### Post-intervention Measures

##### Ability to handle challenges relating to COVID-19

A third question about COVID-19, administered post-intervention only, asked participants if they believed that the content in the program could help them handle challenges relating to coronavirus (“I think the content covered in this program could help me handle challenges related to the coronavirus over the next few weeks.”). This statement was also rated on a 7-point Likert scale ranging from “strongly disagree” to “strongly agree.”

##### Demographic information

Participants were asked to report their biological sex, sexual orientation, race, economic class, and mental health history. To avoid biasing participants’ responses to other questionnaires, we included the demographic questions at the end of the survey. When developing our items and response options, we followed guidelines on best practices for assessing demographic characteristics (see Hughes et al., 2016).

### Analytic Plan

Below, we describe our analyses. All analyses were planned prior to beginning data analysis. Because our primary goal was to examine the potential of the online single-session intervention delivery format (as opposed to specific content modules), we pooled the data from each intervention condition (behavioral activation/cognitive restructuring, behavioral activation/gratitude, and cognitive restructuring/gratitude). Potential differences between conditions will be examined in a future report.

#### Sample Characterization and Usage Patterns

To assess usage patterns with the single-session intervention, we report the number of students who encountered the first screen of their first module (“starters”). Starters are divided into “completers” (people who finished both of the modules assigned to them) and “non-completers” (people who completed only one module or zero modules). Completion rate is the proportion of completers to starters. We also report the number of times the Qualtrics link was clicked. However, because an individual could have clicked the link multiple times, this number does not represent the number of individuals who clicked the link.

We report the symptom levels for completers (*n* = 189) and non-completers (*n* = 74). As demographic information was obtained from participants after they completed both modules, we only have demographic information for completers. Fifteen completers chose not to provide demographic information.

To assess whether completion of intervention was related to reported levels of depressive symptoms, anxiety symptoms, or secondary control, we conducted independent samples *t*-tests comparing completers and non-completers.

#### Acceptability and Perceived Utility

Acceptability was measured after each module. Thus, completers filled out two sets of acceptability ratings (one for each of the two modules they received), while some non-completers (those who completed one module) filled out one set of acceptability ratings. For completers, we averaged the acceptability scores across the two modules that they rated. For non-completers who completed one module, we used the score from the one module they rated. Drawing from previous research on single-session interventions (Schleider et al., 2020), we operationalized mean scores >3 as overall perceived single-session intervention acceptability.

To evaluate potential group-level differences in acceptability ratings, we examined the association between acceptability and sex, race, sexual orientation, age, economic class, depressive symptoms, anxiety symptoms, and secondary control. We also compared acceptability ratings between completers and non-completers.

Helpfulness, engagement, and applicability were also assessed after each module. We treated the ratings by completers and non-completers in the manner described above for acceptability. We report the mean score and standard deviation for each of the three items relating to helpfulness, engagement, and applicability. Because scores were highly correlated (all *r*s > 0.70), we combined these items into a single variable, which we refer to as “perceived utility.” To evaluate potential group-level differences in perceived utility, we conducted the same analyses described above for acceptability.

#### Secondary Control

We computed paired sample *t*-tests and estimated within-group effect sizes (Cohen’s *d* with 95% confidence intervals) to assess reported changes on secondary control from pre-intervention to post-intervention. Following guidelines by Lakens (2013), we calculated the pre–post effect sizes using two methods. We report both *d*_*av*_ (which does not take into account correlations between pre- and post- program measures) and *d*_*z*_ (which accounts for correlations between pre- and post- program measures).

#### Questions About COVID-19

For the two questions about COVID-19 administered pre- and post-intervention, we used the same process described above for secondary control. Because the two COVID-19 questions were conceptually distinct (one asked about ability to handle COVID-related problems and one asked about the perceived impact of COVID-19), we computed separate effect sizes for the two COVID-19 questions.

For the question about the perceived impact of the intervention on participants’ ability to handle coronavirus-related challenges, which was administered post-intervention only, we report the mean, standard deviation, and% endorsement (scores > 4).

#### Missing Data

We used all available data for each analysis described above. Because one of our aims involved examining attrition, missing data are reported but not imputed. Missing data for analyses were handled via pairwise deletion.

## Results

### Sample Characteristics and Usage Patterns

From 3/30/20 to 4/6/20, our survey link received 561 clicks. 263 individuals completed pre-test questions and were assigned to an intervention. Of these, 189 individuals completed both modules of the single-session intervention, yielding an overall completion rate of 72% among those who were assigned to an intervention. Demographic characteristics for completers are presented in Table 1.

**TABLE 1:** Sample demographics.

	***M* (*SD*) or *N* (%)**
*N* (completers)	189 (100%)
PHQ-2	1.98 (1.65)
GAD-2	2.61 (1.86)
Age	31.04 (8.91)
**Race/ethnicity**	
White	114 (66.67%)
Asian	41 (23.98%)
Hispanic/Latinx/Spanish Origin	12 (7.02%)
Black	11 (6.43%)
Middle Eastern or North African	3 (1.75%)
Other	2 (1.17%)
Missing	18
**Sex**	
Female	127 (72.99%)
Male	42 (24.14%)
Other	2 (1.15%)
Prefer not to answer	3 (1.72%)
Missing	15
**Sexual orientation**	
Heterosexual or straight	140 (81.40%)
Bisexual	16 (9.30%)
Queer	10 (5.81%)
Fluid	6 (3.49%)
Gay or Lesbian	5 (2.91%)
Pansexual	5 (2.91%)
Demisexual	3 (1.74%)
Questioning	3 (1.74%)
Prefer not to answer	5 (2.91%)
Missing	17
**Social class (self-reported)**	
Poor	5 (2.89%)
Working class	27 (15.61%)
Middle class	111 (64.16%)
Affluent	30 (17.34%)
Missing	16
**Experienced a mental illness (self-reported)**	
Yes	72 (41.62%)
Unsure	22 (12.72%)
No	79 (45.67%)
Missing	16

**Participants could select multiple options for race and sexual orientation*.*

Qualtrics records the amount of time that individuals spend on the survey. Among completers, the median time spent on the program, inclusive of all questionnaires, was 39.2 min (1st quartile = 26.7 min). However, participants were not prevented from multitasking or taking a break while completing the survey, and they had to complete the questionnaires in addition to the modules. Thus, the Qualtrics figures represent an overestimate of the time required to complete the intervention.

To describe our sample, we provide completers’ and non-completers’ reports of depressive symptoms [completers: *M* = 1.98, *SD* = 1.65; non-completers: *M* = 2.07, *SD* = 1.87; *t*(115.55) = −0.34, *p* = 0.73, *d* = −0.05] and anxiety symptoms [completers: *M* = 2.61, *SD* = 1.86; non-completers: *M* = 2.92, *SD* = 2.03; *t*(119.12) = −1.12, *p* = 0.27, *d* = −0.16]; differences between completers and non-completers were not statistically significant. Applying scoring guidelines for the PHQ-2 (using a cutoff score of 3), 30% of the completers and 36% of non-completers would screen positive for likely clinical depression. Applying scoring guidelines for the GAD-2 (using a cutoff score of 3), 42% of the completers and 47% of non-completers would screen positive for likely clinical anxiety.

### Acceptability and Perceived Utility

Table 2 shows participants’ ratings of acceptability, perceived helpfulness, engagement, and applicability.

**TABLE 2:** Acceptability and feedback ratings on single-session intervention.

	**Completers (*n* = 185) *M* (*SD*)**	**Non-completers with available data (*n* = 24) *M* (*SD*)**
**Acceptability items (range: 1–5)**		
Approve	4.18 (0.68)	4.00 (0.82)
Like	4.14 (0.69)	3.88 (0.90)
Welcome	4.23 (0.69)	3.88 (0.90)
Appeals	4.12 (0.70)	4.00 (0.76)
Average acceptability score	4.17 (0.65)	3.96 (0.79)
**Perceived utility items (range: 1–7)**		
Helpful	5.73 (1.01)	5.52 (1.19)
Engaging	5.57 (1.07)	5.48 (1.08)
Applicable	5.67 (0.97)	5.48 (1.08)
Average perceived utility score	5.66 (0.93)	5.49 (1.06)

We compared ratings on the acceptability metric (the AIM) between completers and non-completers. Because non-completers only had AIM ratings available for one module (the first module they were assigned), we used completers’ scores on their first module for the comparison.

Acceptability ratings are provided for the first module participants received (completers: *M* = 4.14, *SD* = 0.76; non-completers: *M* = 3.96, *SD* = 0.79); differences between completers and non-completers were not statistically significant [*t*(28.86) = 1.09, *p* = 0.29, *d* = 0.24]. For completers, ratings on the first module they completed were not statistically significantly different than ratings on the second module they completed; *M* = 4.19, *SD* = 0.77; *t*(179) = −0.83, *p* = 0.41, *d* = −0.06. 95% of completers and 83% of non-completers provided acceptability ratings that averaged greater than 3.0, with completers more likely to have scores above a 3; *X*^2^(1, *N* = 209) = 4.31, *p* = 0.04.

Differences in acceptability were not statistically significant by sex, race, sexuality, age, economic class, depressive symptoms, or anxiety symptoms (all *p*s > 0.17). There was a weak association between secondary control at baseline and acceptability ratings. Individuals with greater secondary control at baseline reported slightly higher acceptability scores; *r*(207) = 0.21, *p* = 0.002. To investigate this further, we applied a linear regression; we found that a one-point increase on the secondary control scale was associated with an increase on the AIM of 0.08 points. The intercept was 3.69, suggesting that an individual with a score of 0 on the secondary control scale would still be predicted to rate COMET as acceptable. Additionally, at each level of secondary control (ranging from 1 to 9 in our sample), the majority of participants reported an acceptability score greater than three. Thus, while there was a significant association between secondary control and acceptability, even participants reporting lower levels of secondary control tended to view the COMET modules as acceptable.

Participants generally reported favorable ratings (i.e., >4) on the perceived utility items: perceived helpfulness (completers: *M* = 5.73, *SD* = 1.01; non-completers: *M* = 5.52, *SD* = 1.19), engagement (completers: *M* = 5.57, *SD* = 1.07; non-completers: *M* = 5.48, *SD* = 1.08), and applicability (completers: *M* = 5.67, *SD* = 0.97; non-completers: *M* = 5.48, *SD* = 1.08). We also calculated the percentage of participants who endorsed the modules (i.e., mean score > 4) as helpful (90% of completers and 76% of non-completers), engaging (86% of completers and 84% of non-completers), and applicable (89% of completers and 76% of non-completers).

As mentioned, due to the high correlation between these items, we combined these items into one variable (the perceived utility score) to reduce the number of tests we performed. We calculated perceived utility ratings for the first module participants received (completers: *M* = 5.61, *SD* = 1.00; non-completers: *M* = 5.49, *SD* = 1.06); differences between completers and non-completers were not statistically significant [*t*(30.07) = 0.53 *p* = 0.60, *d* = 0.12]. For completers, ratings on the first module they completed were not statistically significantly different than ratings on the second module they completed; *M* = 5.71, *SD* = 1.15; *t*(180) = −1.13, *p* = 0.26, *d* = −0.09. Differences in perceived utility were not statistically significantly different by sex, race, sexuality, age, economic class, depressive symptoms, anxiety symptoms, or completion status (all *p*s > 0.37). Individuals with greater secondary control at baseline reported slightly higher perceived utility scores; *r*(208) = 0.21, *p* = 0.002. The trend described above for acceptability was also found for perceived utility; at each level of secondary control (ranging from 1 to 9 in our sample), the majority of participants reported an acceptability score greater than four. 92% of completers and 84% of non-completers provided perceived utility ratings that averaged greater than 4.0, and there were no statistically significant differences between completers and non-completers; *X*^2^(1, *N* = 210) = 1.67, *p* = 0.20.

### Changes in Secondary Control

Table 3 shows the results of our paired sample t-tests and effect sizes for the measures delivered at both baseline and post-intervention. Completers reported a statistically significant improvement in secondary control from pre-intervention to post-intervention; *t*(173) = −6.53, *p* < 0.001. Secondary control scores were greater post-intervention (*M* = 6.64, *SD* = 1.64) than pre-intervention (*M* = 6.01, *SD* = 1.82), with moderate standardized effect sizes (*d*_*av*_ = 0.36, *d*_*z*_ = 0.50).

**TABLE 3:** Changes from pre-intervention to post-intervention in secondary control and COVID-related questions.

	**Baseline**		**Post-intervention**		**Paired sample *t*-test results**				
***Construct***	***M***	***SD***	***M***	***SD***	***p*-value**	***Mean difference***	***CI***	***d*_*av*_**	***d*_*z*_**
Secondary control (range: 0–9)	6.01	1.82	6.64	1.64	<0.001	0.63	[0.44, 0.82]	0.36	0.50
Negative impact of COVID-19 crisis (range: 1–7)	3.94	1.61	3.60	1.53	<0.001	0.35	[0.15, 0.55]	0.22	0.25
Ability to handle COVID-related lifestyle changes (range: 1–7)	5.50	1.25	5.64	1.05	0.066	0.14	[−0.01, 0.29]	0.13	0.14

### Changes in COVID-19 Questions

Completers reported improvements in the perceived impact of the COVID-19 crisis on their quality of life from pre-intervention to post-intervention. Participants were less likely to endorse the statement that the COVID-19 crisis would have an extremely negative impact on their quality of life post-intervention (*M* = 3.60, *SD* = 1.53) than pre-intervention (*M* = 3.94, *SD* = 1.61), with small standardized effect sizes (*d*_*av*_ = 0.22, *d*_*z*_ = 0.25). This difference was statistically significant; *t*(178) = 3.51, *p* < 0.001. Completers also reported improvements in their perceived ability to handle COVID-related changes from pre-intervention to post-intervention. Participants were slightly more likely to endorse the statement that they would be able to handle COVID-related changes post-intervention (*M* = 5.64, *SD* = 1.05) than pre-intervention (*M* = 5.50, *SD* = 1.25), with small standardized effect sizes (*d*_*av*_ = 0.13, *d*_*z*_ = 0.14). However, this difference did not meet the threshold for statistical significance; *t*(178) = 1.85, *p* = 0.066.

Finally, 88% of completers believed that the content covered in the program could help them manage challenges relating to the COVID-19 crisis (*M* = 5.67, *SD* = 1.17).

## Discussion

Overall, our findings demonstrate that brief online interventions, such as COMET, can be a feasible and useful way to provide support to individuals during the COVID-19 crisis. One major benefit of such interventions is that they are flexible; they can be modified and updated regularly. This is possible even under time constraints; responding to the COVID-19 crisis, our small team was able to adapt an existing online intervention over the course of just 2 weeks. The flexibility of single-session interventions allows them to be rapidly deployed in times of crisis. Once deployed, these interventions can quickly reach large numbers of people. In just 1 week, 263 individuals began and 189 individuals completed COMET. We not only found that many graduate and professional students were interested in the single-session intervention and willing to complete it, but also that they rated COMET highly on acceptability and perceived utility. Furthermore, acceptability ratings and perceived utility ratings did not differ by reported depressive symptoms or anxiety symptoms; COMET appears to be welcomed by individuals with elevated mental health symptoms as well as those without. Acceptability ratings differed slightly by secondary control. Individuals with higher secondary control–the sense that they can control their reactions to objective circumstances–tended to provide slightly higher ratings of acceptability. This finding might be explained by the fact that individuals who believe they can handle their own responses may be more likely to like programs that promote agency in responding to objective circumstances.

Our findings also provide preliminary data suggesting that these interventions can be helpful during times of crisis. Participants reported greater levels of secondary control post-intervention, which may be especially important during the COVID-19 crisis. Perceived primary control (the belief individuals can influence objective events and circumstances in their life) and secondary control are protective factors for the development of mental health problems (Rothbaum et al., 1982; Weisz et al., 2010). However, while individuals have some control over the protective measures they take against COVID-19 (such as staying in their homes and avoiding gatherings), the crisis has limited the ability of people to influence their objective social, emotional, academic, and economic circumstances. COVID-19 has made it difficult or impossible for individuals to safely visit their loved ones, protect those who are exposed to the virus, keep their jobs, and maintain their daily routines. More broadly, individuals have little control over how long the pandemic will last, how the economy will change, or how the virus will affect the health and lives of their loved ones. In this context, we believe that secondary control, the tendency to believe that one can cope with stressful situations even when one has little control over the outcomes, will be especially important during the crisis. Interventions that improve perceived secondary control will be essential public health tools in the months ahead.

As we have emphasized, it is also important to evaluate the acceptability, implementation, and uptake of such interventions. Unfortunately, as mentioned previously, few digital interventions have demonstrated acceptability and uptake in real-world settings (Fleming et al., 2018; Buss et al., 2020). Drop-out rates in open trials of digital interventions are high (Fleming et al., 2018), users rarely spend more than a few minutes on digital mental health interventions (Baumel et al., 2019), and most publicly available mental health apps generally fail to retain users (Wasil et al., 2020e). With this in mind, some of our findings are especially promising. Individuals enrolled in our intervention over a short timeframe, considered our intervention acceptable, and believed our intervention was helpful, engaging, and applicable to their lives. Identifying specific strategies that led to high acceptability, especially strategies that could be replicated in future mental health promotion efforts, could be highly valuable.

There are a few unique aspects of our process that may have led to favorable acceptability ratings and uptake. First, we intentionally advertised COMET as a program that all students could benefit from. Drawing from work in low- and middle-income countries (e.g., Osborn et al., 2019), we reasoned that branding our intervention as a program that anyone could benefit from could circumvent some of the stigma associated with help-seeking for psychiatric disorders. Consequently, rather than mentioning depression or anxiety, we branded the intervention as one that could help individuals “adjust to changing life circumstances, manage emotions, and achieve goals”. Furthermore, rather than referring to two of the modules as “behavioral activation” and “cognitive restructuring”–technical terms that may be associated with formal psychotherapy–we relabeled these sections as “positive activities” and “flexible thinking.” Future research could examine different ways to present and advertise these or other evidence-based intervention modules. Such research could draw from work on the direct-to-consumer marketing of mental health interventions (Becker, 2015; Rith-Najarian et al., 2019b).

Our partnership with university deans was also essential. As a result of this partnership, information about our intervention was distributed to a wide array of students across the university. Additionally, since the recruitment messages were sent out using the official student listservs (which are often used for important communications), students may have been more likely to notice and open the message. In these ways, our findings showcase the potential utility of partnerships between researchers and university administrators. Finally, it is important to note that the COVID-19 crisis may have impacted our recruitment efforts. Students may be especially interested in developing skills that can help them cope with lifestyle changes as a result of the crisis. Additionally, due to social distancing and online learning, some students may have more free time than they normally do during the semester, which may have made them more responsive to our web-based intervention.

Our findings also have implications for public health officials, higher education leaders, and intervention developers interested in supporting people during the COVID-19 crisis. For example, our promising findings regarding students’ experiences with COMET could encourage future collaborations between psychologists and higher education leaders. While such partnerships can be time-consuming and effortful, they are worthwhile to pursue when there is reason to believe that they will be impactful. This is especially true for collaborations around topics that could be considered sensitive, like programs relating to mental health. When we launched our collaboration, our team and our collaborators did not know if or how students would engage with our program. Our experience offers some room for optimism, showing that such collaborations can be fruitful, and students appear highly receptive to university-endorsed online mental health initiatives. With this in mind, we hope our findings encourage psychologists and higher education leaders at other universities to engage in student mental health promotion initiatives during the COVID-19 crisis. Individuals considering such collaborations or interested in developing interventions may benefit from adapting content from pre-existing interventions or common elements from empirically supported interventions (see Chorpita and Daleiden, 2009; Weisz et al., 2012). In our experience, the decision to adapt existing modules (rather than to create a new intervention from scratch) allowed us to act quickly while ensuring that individuals received content with strong empirical support. From a public health perspective, repurposing existing interventions may provide a quick and efficient way to expand access to support. Additionally, modular interventions may be especially valuable given their flexibility and adaptability (Weisz et al., 2012). As an example, COMET was adapted for graduate and professional students over the course of a few weeks. COMET, or other modular interventions, could also be readily adapted for additional populations. New modules (e.g., mindfulness and problem solving) could be added, existing modules could be removed, or different combinations of modules could be deployed depending on the needs of specific populations. Finally, our findings highlight that students of a variety of age groups and backgrounds are interested in online self-help interventions. With this in mind, higher education leaders could consider launching low-intensity interventions to support students across the country.

Our findings should be interpreted in light of some limitations. First, our pre-post design is not sufficient to make causal claims, and our study does not remove the need for randomized control trials. It remains unknown if our intervention can produce lasting changes in participants’ thoughts, behaviors, or feelings. In order to gauge those effects, there is a need for adequately-powered pre-registered randomized controlled trials which measure mental health outcomes (e.g., depressive and anxiety symptoms) longitudinally. Second, the rates of depressive symptoms and anxiety symptoms identified in our study should be interpreted within the context of COVID-19. Participants’ reports of depression and anxiety may be higher than normal due to the stressors and lifestyle changes introduced by the crisis. For some individuals, these symptoms may be temporary, but for others, they may last beyond the crisis; future longitudinal and observational research would be useful to examine these trends. Third, while our sample was diverse along several dimensions, participants in our study were predominantly female. This is consistent with previous research; a recent review documented that most studies of prevention programs for college students and graduate students had samples that were two-thirds or more female (Rith-Najarian et al., 2019a). Such findings call for the development of recruitment techniques that may make digital interventions more appealing to male students (e.g., Rith-Najarian et al., 2019b). Fourth, in order to minimize participant burden and maximize the reach of our survey, our demographic questionnaire was brief. As a result, we did not comprehensively assess contextual factors that may be highly relevant during the COVID-19 crisis, such as participants’ living situations, income, marital status, social support, or parental status. Future research is needed to understand contextual risk factors and protective factors that may influence how students are affected by COVID-19. Additionally, future research could evaluate if psychosocial interventions can support students who are especially vulnerable during the crisis. Finally, participants in our study received pre-program and post-program questionnaires before being assigned to an intervention condition, making it difficult to estimate the exact amount of time that participants spent completing intervention content Furthermore, it is possible that our baseline questionnaires deterred some individuals from engaging. If our intervention had not included baseline questionnaires, it is possible that it would have reached more students.

Overall, our findings suggest that brief digital interventions could be a useful way to expand access to care in times of public health emergencies such as the COVID-19 crisis. Students appear interested in these interventions, complete them at high rates, and find them helpful. Participants also reported improvements in their perceived sense of control and ability to handle the pandemic from pre- to post-intervention. Future research is needed to understand which content is best suited for brief interventions, which content is best suited for specific circumstances (e.g., public health emergencies), how such interventions should be ideally presented and disseminated, and for whom these interventions are most effective. Such research could ensure that the important findings and interventions from psychological science are successfully disseminated to the broader public, especially during public health emergencies like the COVID-19 pandemic.

## Data Availability Statement

The datasets generated for this study are not readily available because the authors did not receive clearance to share the data collected for this project. The R code associated with this project has been made available as a Supplementary Material. Requests to access the datasets should be directed to AW, wasil@sas.upenn.edu.

## Ethics Statement

The studies involving human participants were reviewed and approved by University of Pennsylvania Institutional Review Board. Written informed consent for participation was not required for this study in accordance with the national legislation and the institutional requirements.

## Author Contributions

AW conceptualized the idea for the study with RD. AW and MT designed the initial versions of the interventions with guidance from RD. RF and JS participated in the adaptation of the interventions. AW performed data analysis with oversight from RD and support from MT, RF, and JS. AW wrote the initial R script. RF, MT, and JS reviewed and revised the script. AW wrote the initial draft of the manuscript. MT, RF, JS, and RD revised the manuscript. All authors contributed to the article and approved the submitted version.

## Conflict of Interest

The authors declare that the research was conducted in the absence of any commercial or financial relationships that could be construed as a potential conflict of interest.

## References

[B1] Baumel A. Muench F. Edan S. Kane J. M. (2019). Objective user engagement with mental health apps: systematic search and panel-based usage analysis. *J. Med. Internet Res.* 21:e14567. 10.2196/14567 PMC6785720 31573916

[B2] Becker S. J. (2015). Direct-to-consumer marketing: a complementary approach to traditional dissemination and implementation efforts for mental health and substance abuse interventions. *Clin. Psychol. Sci. Pract.* 22 85–100. 10.1111/cpsp.12086 PMC4415980 25937710

[B3] Buss J. Lorenzo-Luaces L. Banks G. Horani D. Rutter L. Wasil A. (2020). Availability of internet Cognitive Behavioral Therapies (iCBTs). *PsyArXiv* [Preprint]. 10.31234/osf.io/2jwgd PMC10787155 38216233

[B4] Chirikov I. Soria K. M. Horgos B. Jones-White D. (2020). *Undergraduate and Graduate Students’ Mental Health during the COVID-19 Pandemic. SERU Consortium, University of California - Berkeley and University of Minnesota.* Available online at: https://cshe.berkeley.edu/seru-covid-survey-reports (accessed March 10, 2021).

[B5] Chorpita B. F. Daleiden E. L. (2009). Mapping evidence-based treatments for children and adolescents: application of the distillation and matching model to 615 treatments from 322 randomized trials. *J. Consult. Clin. Psychol.* 77 566–579. 10.1037/a0014565 19485596

[B6] Cregg D. R. Cheavens J. S. (2020). Gratitude interventions: effective self-help? A meta-analysis of the impact on symptoms of depression and anxiety. *J. Happiness Stud.* 22 413–445. 10.1007/s10902-020-00236-6

[B7] Davis D. E. Choe E. Meyers J. Wade N. Varjas K. Gifford A. (2016). Thankful for the little things: a meta-analysis of gratitude interventions. *J. Counsel. Psychol.* 63 20–31. 10.1037/cou0000107 26575348

[B8] Dickens L. R. (2017). Using gratitude to promote positive change: a series of meta-analyses investigating the effectiveness of gratitude interventions. *Basic Appl. Soc. Psychol.* 39 193–208. 10.1080/01973533.2017.1323638

[B9] Evans T. M. Bira L. Gastelum J. B. Weiss L. T. Vanderford N. L. (2018). Evidence for a mental health crisis in graduate education. *Nat. Biotechnol.* 36 282–284. 10.1038/nbt.4089 29509732

[B10] Fleming T. Bavin L. Lucassen M. Stasiak K. Hopkins S. Merry S. (2018). Beyond the trial: systematic review of real-world uptake and engagement with digital self-help interventions for depression, low mood, or anxiety. *J. Med. Internet Res.* 20:e199. 10.2196/jmir.9275 PMC6010835 29875089

[B11] Higa-McMillan C. K. Francis S. E. Rith-Najarian L. Chorpita B. F. (2016). Evidence base update: 50 years of research on treatment for child and adolescent anxiety. *J. Clin. Child Adolesc. Psychol.* 45 91–113. 10.1080/15374416.2015.1046177 26087438

[B12] Hofmann S. G. Hayes S. C. (2019). The future of intervention science: process-based therapy. *Clin. Psychol. Sci.* 7 37–50. 10.1177/2167702618772296 30713811 PMC6350520

[B13] Hughes J. Camden A. Yangchen T. (2016). Rethinking and updating demographic questions: guidance to improve descriptions of research samples. *PSI CHI J. Psychol. Res.* 21 138–151. 10.24839/2164-8204.JN21.3.138

[B14] Josephine K. Josefine L. Philipp D. David E. Harald B. (2017). Internet- and mobile-based depression interventions for people with diagnosed depression: a systematic review and meta-analysis. *J. Affect. Disord.* 223 28–40. 10.1016/j.jad.2017.07.021 28715726

[B15] Karyotaki E. Riper H. Twisk J. Hoogendoorn A. Kleiboer A. Mira A. (2017). Efficacy of self-guided internet-based cognitive behavioral therapy in the treatment of depressive symptoms: a meta-analysis of individual participant data. *JAMA Psychiatry* 74 351–359. 10.1001/jamapsychiatry.2017.0044 28241179

[B16] Kazdin A. E. Blase S. L. (2011). Rebooting psychotherapy research and practice to reduce the burden of mental illness. *Perspect. Psychol. Sci.* 6 21–37. 10.1177/1745691610393527 26162113

[B17] Kroenke K. Spitzer R. L. Williams J. B. W. (2003). The patient health questionnaire-2: validity of a two-item depression screener. *Med. Care* 41 1284–1292. 10.1097/01.MLR.0000093487.78664.3C 14583691

[B18] Kroenke K. Spitzer R. L. Williams J. B. W. Monahan P. O. Löwe B. (2007). Anxiety disorders in primary care: prevalence, impairment, comorbidity, and detection. *Ann. Intern. Med.* 146 317–325. 10.7326/0003-4819-146-5-200703060-00004 17339617

[B19] Lakens D. (2013). Calculating and reporting effect sizes to facilitate cumulative science: a practical primer for t-tests and ANOVAs. *Front. Psychol.* 4:863. 10.3389/fpsyg.2013.00863 PMC3840331 24324449

[B20] Liu S. Yang L. Zhang C. Xiang Y.-T. Liu Z. Hu S. (2020). Online mental health services in China during the COVID-19 outbreak. *Lancet Psychiatry* 7 e17–e18. 10.1016/S2215-0366(20)30077-8 32085841 PMC7129099

[B21] Mohr D. C. Lyon A. R. Lattie E. G. Reddy M. Schueller S. M. (2017). Accelerating digital mental health research from early design and creation to successful implementation and sustainment. *J. Med. Internet Res.* 19:e153. 10.2196/jmir.7725 PMC5443926 28490417

[B22] Murray L. K. Dorsey S. Haroz E. Lee C. Alsiary M. M. Haydary A. (2014). A common elements treatment approach for adult mental health problems in low- and middle-income countries. *Cogn. Behav. Pract.* 21 111–123. 10.1016/j.cbpra.2013.06.005 25620867 PMC4304666

[B23] Nathan P. E. Gorman J. M. (2015). *A Guide to Treatments that Work*, 4th Edn. Oxford: Oxford University Press, xxvii, 956.

[B24] Osborn T. L. Rodriguez M. Wasil A. R. Venturo-Conerly K. E. Gan J. Alemu R. G. (2020). Single-session digital intervention for adolescent depression, anxiety, and well-being: outcomes of a randomized controlled trial with Kenyan adolescents. *J. Consult. Clin. Psychol.* 88 657–668. 10.1037/ccp0000505 32391709

[B25] Osborn T. L. Wasil A. R. Venturo-Conerly K. E. Schleider J. L. Weisz J. R. (2019). Group intervention for adolescent anxiety and depression: outcomes of a randomized trial with adolescents in Kenya. *Behav. Ther.* 51 601–615. 10.1016/j.beth.2019.09.005 32586433

[B26] Pfefferbaum B. North C. S. (2020). Mental health and the Covid-19 pandemic. *N. Engl. J. Med.* 383 510–512. 10.1056/NEJMp2008017 32283003

[B27] Plummer F. Manea L. Trepel D. McMillan D. (2016). Screening for anxiety disorders with the GAD-7 and GAD-2: a systematic review and diagnostic metaanalysis. *Gen. Hosp. Psychiatry* 39 24–31. 10.1016/j.genhosppsych.2015.11.005 26719105

[B28] Rith-Najarian L. R. Boustani M. M. Chorpita B. F. (2019a). A systematic review of prevention programs targeting depression, anxiety, and stress in university students. *J. Affect. Disord.* 257 568–584. 10.1016/j.jad.2019.06.035 31326690

[B29] Rith-Najarian L. R. Sun W. Chen A. Chorpita B. Chavira D. Mougalian S. (2019b). What’s in a name? Branding of online mental health programming for university students. *J. Consult. Clin. Psychol.* 87 380–391. 10.1037/ccp0000383 30883165

[B30] Rothbaum F. Weisz J. R. Snyder S. S. (1982). Changing the world and changing the self: a two-process model of perceived control. *J. Pers. Soc. Psychol.* 42 5–37. 10.1037/0022-3514.42.1.5

[B31] Sahu P. (2020). Closure of universities due to coronavirus disease 2019 (COVID-19): impact on education and mental health of students and academic staff. *Cureus* 12:e7541. 10.7759/cureus.7541 PMC7198094 32377489

[B32] Schleider J. Weisz J. (2018). A single-session growth mindset intervention for adolescent anxiety and depression: 9-month outcomes of a randomized trial. *J. Child Psychol. Psychiatry* 59 160–170. 10.1111/jcpp.12811 28921523

[B33] Schleider J. L. Dobias M. Sung J. Mumper E. Mullarkey M. C. (2020). Acceptability and utility of an open-access, online single-session intervention platform for adolescent mental health. *JMIR Ment. Health* 7:e20513. 10.2196/20513 PMC7367540 32602846

[B34] Schleider J. L. Weisz J. R. (2017). Little treatments, promising effects? Meta-analysis of single-session interventions for youth psychiatric problems. *J. Am. Acad. Child Adolesc. Psychiatry* 56 107–115. 10.1016/j.jaac.2016.11.007 28117056

[B35] Seligman M. E. P. Steen T. A. Park N. Peterson C. (2005). Positive psychology progress: empirical validation of interventions. *Am. Psychol.* 60 410–421. 10.1037/0003-066X.60.5.410 16045394

[B36] Torous J. Jän Myrick K. Rauseo-Ricupero N. Firth J. (2020). Digital mental health and COVID-19: using technology today to accelerate the curve on access and quality tomorrow. *JMIR Ment. Health* 7:e18848. 10.2196/18848 PMC7101061 32213476

[B37] Torous J. Nicholas J. Larsen M. E. Firth J. Christensen H. (2018). Clinical review of user engagement with mental health smartphone apps: evidence, theory and improvements. *Evid. Based Ment. Health* 21 116–119. 10.1136/eb-2018-102891 29871870 PMC10270395

[B38] Walton G. M. Wilson T. D. (2018). Wise interventions: psychological remedies for social and personal problems. *Psychol. Rev.* 125 617–655. 10.1037/rev0000115 30299141

[B39] Wasil A. R. Franzen R. E. Gillespie S. Steinberg J. S. Malhotra T. DeRubeis R. J. (2021a). Commonly reported problems and coping strategies during the COVID-19 crisis: a survey of graduate and professional students. *Front. Psychol.* 12:598557. 10.3389/fpsyg.2021.598557 PMC7947789 33716864

[B40] Wasil A. R. Gillespie S. Patel R. Petre A. Venturo-Conerly K. E. Shingleton R. M. (2020a). Reassessing evidence-based content in popular smartphone apps for depression and anxiety: developing and applying user-adjusted analyses. *J. Consult. Clin. Psychol.* 88 983–993. 10.1037/ccp0000604 32881542

[B41] Wasil A. R. Gillespie S. Schell T. Lorenzo−Luaces L. DeRubeis R. J. (2021b). Estimating the real−world usage of mobile apps for mental health: development and application of two novel metrics. *World Psychiatry* 20 137–138. 10.1002/wps.20827 33432761 PMC7801857

[B42] Wasil A. R. Gillespie S. Shingleton R. Wilks C. R. Weisz J. R. (2020b). Examining the reach of smartphone apps for depression and anxiety. *Am. J. Psychiatry* 177 464–465. 10.1176/appi.ajp.2019.19090905 32354266

[B43] Wasil A. R. Kacmarek C. N. Osborn T. L. Palermo E. H. DeRubeis R. J. Weisz J. R. (2020c). Economic evaluation of an online single-session intervention for depression in Kenyan adolescents. *PsyArXiv* [Preprint]. 10.31234/osf.io/qrafg 34472893

[B44] Wasil A. R. Park S. J. Gillespie S. Shingleton R. Shinde S. Natu S. (2020d). Harnessing single-session interventions to improve adolescent mental health and well-being in India: development, adaptation, and pilot testing of online single-session interventions in Indian secondary schools. *Asian J. Psychiatry* 50:101980. 10.1016/j.ajp.2020.101980 32146337

[B45] Wasil A. R. Patel R. Cho J. Shingleton R. M. Weisz J. R. DeRubeis R. J. (2021c). Smartphone apps for eating disorders: a systematic review of evidence-based content and application of user-adjusted analyses. *Int. J. Eating Disord.* 10.1002/eat.23478 33534176

[B46] Wasil A. R. Venturo-Conerly K. E. Shingleton R. M. Weisz J. R. (2019). A review of popular smartphone apps for depression and anxiety: assessing the inclusion of evidence-based content. *Behav. Res. Ther.* 123:103498. 10.1016/j.brat.2019.103498 31707224

[B47] Wasil A. R. Weisz J. R. DeRubeis R. J. (2020e). Three questions to consider before developing a mental health app. *World Psychiatry* 19 252–253. 10.1002/wps.20757 32394568 PMC7215053

[B48] Weiner B. J. Lewis C. C. Stanick C. Powell B. J. Dorsey C. N. Clary A. S. (2017). Psychometric assessment of three newly developed implementation outcome measures. *Implement. Sci.* 12:108. 10.1186/s13012-017-0635-3 PMC5576104 28851459

[B49] Weisz J. R. Chorpita B. F. Palinkas L. A. Schoenwald S. K. Miranda J. Bearman S. K. (2012). Testing standard and modular designs for psychotherapy treating depression, anxiety, and conduct problems in youth: a randomized effectiveness trial. *Arch. Gen. Psychiatry* 69 274–282. 10.1001/archgenpsychiatry.2011.147 22065252

[B50] Weisz J. R. Francis S. E. Bearman S. K. (2010). Assessing secondary control and its association with youth depression symptoms. *J. Abnorm. Child Psychol.* 38 883–893. 10.1007/s10802-010-9440-z 20640503 PMC3001242

[B51] Zhai Y. Du X. (2020). Addressing collegiate mental health amid COVID-19 pandemic. *Psychiatry Res.* 288:113003. 10.1016/j.psychres.2020.113003 PMC7162776 32315885

