# Supplementary material for: Promoting Graduate Student Mental Health During COVID-19: Acceptability, Feasibility, and Perceived Utility of an Online Single-Session Intervention
Source: Front Psychol. 2021 Apr 7;12:569785. doi: 10.3389/fpsyg.2021.569785 (PMC8058455; doi:10.3389/fpsyg.2021.569785)
Supplement: Supplementary file 1 [file Table_1.DOCX]

**Supplement 1: Development of COMET**

In this file, we describe the procedures our study team followed when developing the *Common Elements Toolbox* (COMET). While our core procedures are described in the manuscript, this supplemental file is intended to provide greater detail into our process. We have also included supplemental figures showing examples of the exercises in our intervention. We hope this document is useful to individuals interested in developing brief online mental health and wellness interventions.

For additional details about the procedure or the intervention, please contact the corresponding author at [wasil@sas.upenn.edu](mailto:wasil@sas.upenn.edu).

**Overview**

In the fall of 2019, we became interested in seeing if online single-session interventions could be a useful way to promote mental health and wellness among American college students. We were inspired by research which suggested that brief, self-guided, single-session interventions could reduce depression and anxiety (Schleider & Weisz, 2017; Schleider & Weisz, 2018) and previous work on single-session interventions that our team had conducted with adolescents in India (Wasil et al., 2020). We were also guided by research on the advantages of modular interventions (Murray et al., 2014; Weisz et al., 2012) based on common elements from empirically supported interventions (Chorpita & Daleiden, 2009; Higa-McMillan et al., 2016).

As a result, we planned to develop and evaluate an online single-session intervention for undergraduate students via a randomized controlled trial. We planned this study in the fall of 2019, before we knew that a pandemic was imminent. Currently, the pre-registered randomized controlled trial (NCT04287374) is underway and data collection is scheduled to end in August of 2020. We initially had no plans to release a version of the intervention for graduate and professional students. However, in light of the coronavirus disease 2019 (COVID-19) pandemic, we decided to adapt the intervention for graduate and professional students and tailor the content to address stressors they may experience during COVID-19.

Below, we detail our procedures over the course of fall 2019 to spring 2020 to a) develop a version of COMET for undergraduate students, b) prepare a randomized controlled trial to test the intervention, and c) adapt the existing intervention for graduate and professional students.

**Developing a Version of COMET for Undergraduate Students (before COVID-19)**

**Selection of Intervention Modules**

Our first decision involved selecting the modules to include in our intervention. We surveyed previous research to identify common intervention procedures in empirically supported treatments (Chorpita & Daleiden, 2009; Higa-McMillan et al., 2016; Murray et al., 2014; Nathan & Gorman, 2015; Weisz et al., 2012), existing digital interventions (Wasil et al., 2019), single-component wise interventions (Schleider, Mullarkey & Chacko, 2020; Walton, 2014; Walton & Wilson, 2018), and positive psychology interventions (Chaves et al., 2017; Taylor, Lyubomirsky, & Stein, 2017; Schueller, 2010; Rashid & Seligman, 2018; Seligman et al., 2005; Seligman et al., 2006). Then, we consulted with licensed clinical psychologists (to discuss which modules might be best suited for an online self-help format) and conducted interviews with undergraduate students (to gauge their perceptions of the appeal and utility of different intervention modules). Through this process, we selected three modules with empirical support and potential appeal: behavioral activation, cognitive restructuring, and gratitude.

**Development of Intervention Modules**

After selecting the modules, we developed initial drafts of each intervention module using the online software Qualtrics. The design and layout of the intervention was informed by previous research on single-session interventions (Schleider et al., 2018; Schleider et al., 2020) and a review of commercially available smartphone-based interventions (Wasil et al., 2019). We sought to design intervention modules that are simple, straightforward, clear, and that minimize participant burden. Each intervention was developed in consultation with RJD (a licensed clinical psychologist and Director of Clinical Training at the University of Pennsylvania with experience delivering in-person cognitive behavioral therapy) and Penn undergraduate students. Below, we describe the sources that informed the development of each module.

***Behavioral Activation***

To develop the behavioral activation (“positive activities”) module, we drew from previous research on behavioral activation interventions (Dimidjian et al., 2006; Patel et al., 2017; Schleider, Dobias, et al., 2020). Our team also surveyed two online behavioral activation interventions for adolescents: the Action Brings Change intervention (ABC Project; Schleider et al., 2019) and an intervention we previously developed for Indian adolescents (Wasil et al., 2020). Because these previous interventions were developed for adolescents, we consulted with undergraduate students in our lab to assess their potential suitability for college students. The undergraduate students believed that both of these existing intervention templates were too long to be included in a three-component intervention, included several introductory reading passages that would not be necessary for a college audience, used language and examples tailored to a younger audience, and did not focus enough on interactive exercises. Based on their perspectives, and our own judgments about the relevance of the previous interventions for undergraduate students, we decided not to use the ABC Project or our previous intervention as the template for the intervention we were developing. Instead, we reasoned that our intervention would be more impactful if we designed it from scratch, incorporating feedback from undergraduates at every step. Thus, after reviewing the aforementioned sources, we designed an intervention module in which participants a) briefly learn about pleasurable activities and mastery activities, b) identify (in their own words) pleasurable activities and mastery activities that they wanted to perform more frequently in the next few weeks, c) choose one activity to commit to performing more frequently, and d) reflect on why the activity they selected is important to them.

***Cognitive Restructuring***

To develop the cognitive restructuring (“flexible thinking”) module, we reviewed worksheets that are commonly included as homework assignments in cognitive therapies (e.g., [https://bit.ly/2SZwxso](https://www.google.com/url?q=https://bit.ly/2SZwxso&sa=D&ust=1589325991714000&usg=AFQjCNH85yKbAW2TL0QmLrbxIcmc29ngUQ)). We also relied on guidance from RJD, an expert with extensive experience studying and delivering cognitive therapy. Because cognitive restructuring can be somewhat complicated to learn for the first time, we reasoned that students may find it helpful to apply this technique to a hypothetical student before applying it to themselves. Using this approach, participants could acquire experience in using the technique by applying it to a standardized example. Additionally, this approach also allowed them to receive feedback to ensure that they understood how to use the technique. After a participant applied each step of the cognitive restructuring technique to the hypothetical student, we showed the student an example response (see Figure S3). We also thought this approach could be desirable given that “saying-is-believing” exercises are thought to be helpful in previous single-session interventions (e.g., Schleider & Weisz, 2018). Thus, we designed a cognitive restructuring module in which participants a) read about another student experiencing distress, b) apply a standard cognitive restructuring technique to that student’s life, and c) apply the same technique to a negative situation in their own life.

***Gratitude***

To develop the gratitude module, we surveyed gratitude exercises included in empirically supported positive psychology interventions (Emmons & Stern; 2013; Kushlev et al., 2017; Schueller, 2010; Seligman et al., 2005; Seligman et al., 2006; Rashid & Seligman, 2018; Taylor et al., 2016). We also drew from the gratitude module of a previous intervention for Kenyan adolescents that the first author developed with collaborators (Osborn et al., 2020). As a result, we developed a gratitude module in which participants a) write about and reflect on three good things that have happened recently (adapted from Seligman et al., 2005) and b) develop an awareness of things they can be grateful for in their physical environment.

**Refinement of Intervention Modules**

After developing the initial drafts of each intervention module, we refined the intervention by soliciting feedback from colleagues and undergraduate students. We performed a series of semi-structured interviews in which participants completed an intervention module while we asked them questions about their perception of the intervention’s clarity, engagement, and potential helpfulness. The purpose of these interviews was to understand undergraduate students’ perceptions about the intervention and receive recommendations we could use to improve the interventions.

As a result of this process, we made several stylistic changes to the intervention modules. These changes generally involved reducing the amount of text in explanatory sections and modifying content to make the intervention more engaging for participants. For instance, we shortened the length of each module, reduced the amount of text per page, included more color and pictures, and increased the font size on several pages. These changes were made during fall 2019.

**Conducting a Randomized Controlled Trial of COMET for Undergraduate Students**

As we finalized this intervention (which was later named COMET), we designed a randomized controlled trial to evaluate its impact on undergraduate student mental health and well-being. This trial was registered on clinicaltrials.gov (NCT04287374). We began data collection for this trial in March 2020. Data collection is still underway and is expected to be completed in August 2020.

For the randomized controlled trial, participants (undergraduate students at the University of Pennsylvania or Harvard University) were randomized to receive COMET (all three modules) or a control condition teaching study skills. Participants were instructed to fill out measures of depressive symptoms, anxiety symptoms, and subjective well-being at baseline and three follow-up timepoints: two weeks post-intervention, four weeks post-intervention, and twelve weeks post-intervention. Participants also received financial compensation or course credit for their participation. The results of this randomized controlled trial will allow us to evaluate the impact of COMET on mental health outcomes relative to a study skills control condition.

**Adaptation of COMET for Graduate Students (during COVID-19)**

As we were launching the randomized controlled trial for undergraduate students, the COVID-19 crisis began to affect students at our university. In late March, the University of Pennsylvania announced that all classes and non-essential research activities would be moved online. This decision, while important to prevent the spread of the disease, resulted in considerable uncertainty, stress, and lifestyle changes for students. To support students during this difficult transition period, we teamed up with the Behavior Change for Good Initiative, an interdisciplinary group of academic experts that regularly performs large-scale field experiments (see <https://bcfg.wharton.upenn.edu/>). In partnership with the Behavior Change for Good Initiative, we contacted university deans to gauge their interest in a collaboration to use elements of COMET to support students during the crisis. We had already released the version of COMET for undergraduate students (while recruiting for our randomized controlled trial), and our university partners believed our program could be especially well-suited for graduate and professional students during the crisis. As a result, we decided to adapt the undergraduate version of COMET for graduate and professional students. The deans agreed to help us by reviewing our intervention materials and disseminating the intervention to graduate and professional students. After receiving the support of the deans, we then began to adapt our intervention for graduate and professional students at Penn.

As we planned this project, we worked with the Behavior Change for Good Institute to submit a Quality Improvement/Quality Assurance (QI/QA) Project Determination Form to the University of Pennsylvania Institutional Review Board. The form included a summary of our design, the data collection methods we planned to employ, background for the project, and a description of how the project would be used to assess and improve behavior change interventions. QI/QA determination is based on a variety of factors including the purpose of the study, the study design (e.g., does the study include a control group?), procedures (e.g., whether or not the interventions are evidence-based), populations, risks to participants beyond standard care/practice, funding, personnel (e.g., Penn employees or external agencies), and applicability of findings (see <https://irb.upenn.edu/mission-institutional-review-board-irb/guidance/qiqa-project-guidance>). We reasoned that our study would be eligible for QI/QA determination because a) we were not assigning participants to a control group, b) we were using evidence-based intervention modules, c) the project posed minimal risks to participants, and d) the project was conducted by Penn employees for Penn students. We received QI/QA approval on March 23, 2020.

With the support of university deans and Behavior Change for Good researchers, we adapted our intervention for graduate and professional students. This adaptation, which occurred over two weeks, shortened the intervention, increased its relevance for graduate students, and increased its relevance to challenges during the COVID-19 crisis. Our first step involved deciding how many modules each participant would receive. When making this choice, we were guided by three objectives: a) providing evidence-based content to participants, b) minimizing participant burden, and c) incorporating the advice of university deans. Initially, we planned to provide all three modules to participants. We reasoned that this approach would maximize the chance that any given participant would benefit from the intervention. However, when we consulted with university deans about this decision, they informed us that they believed the reach and impact of the intervention would be greater if we reduced its length. Specifically, they predicted that participants would be willing to spend about 20-25 minutes on intervention content. Based on pilot data that we had collected during our interviews with undergraduate students, we expected that each module would take approximately 10-12 minutes. Therefore, we reasoned that providing two modules to each participant would keep the intervention at an appropriate length while still offering individuals access to multiple modules.

Next, members of the study team and students in our lab produced a modified version of the intervention modules in Qualtrics. Then, each member of the study team independently reviewed the intervention and generated feedback. After we made an additional round of edits to the intervention, we sent the intervention to a group of scholars affiliated with the Behavior Change for Good Institute to solicit additional feedback. Their feedback focused on ways to improve the intervention’s design and engagement. After we incorporated their feedback, two university deans reviewed the intervention to gauge its appropriateness and suitability for graduate students. After addressing the deans’ comments, we informally piloted the intervention with several colleagues including graduate students, licensed clinical psychologists, professors, and non-academics. In total, about 20 individuals outside of our research team reviewed the intervention. These individuals primarily gave feedback on the intervention design (e.g., font sizes, colors) and detected any grammatical or formatting errors (e.g., typos, spacing problems). Finally, our core research team re-reviewed the final version of the intervention prior to its dissemination. On March 30, 2020, we released the intervention to graduate students and professional students over university listservs. The intervention was sent out on official listservs for students in the Graduate School of Arts and Sciences (<https://www.sas.upenn.edu/>) and the College of Liberal and Professional Studies (<https://www.sas.upenn.edu/lps/>).

We took steps to reduce the potential for error during our data analysis process. First, to catch potential errors with downloading the dataset and recoding variables, two members of the research team independently downloaded the dataset and recoded variables. When these two datasets were compared, they were found to be identical. Furthermore, four members of our research team independently reviewed the R code used for the data analyses. Finally, we included a version of our R code as a supplemental file in this submission.

**Conclusion**

Overall, we believe that both studies—the randomized trial with undergraduates and the uncontrolled acceptability study with graduate and professional students—will meaningfully advance our understanding of brief digital mental health promotion interventions. Through the randomized trial, in which participants were compensated for participation, we will be able to understand the impact of COMET on mental health and wellness outcomes. Through the uncontrolled study, in which participants had no extrinsic incentive to participate, we have been able to understand the acceptability, uptake, and implementation of COMET in a naturalistic real-world setting. Future research is needed in both areas (efficacy and acceptability) to improve the impact and reach of digital mental health promotion interventions. Such research will be especially impactful during times of crisis, when timely evidence-based support is most needed.

**Supplemental Figures: Examples of COMET Exercises**

Figure S1

*Forming a Positive Activity Plan*


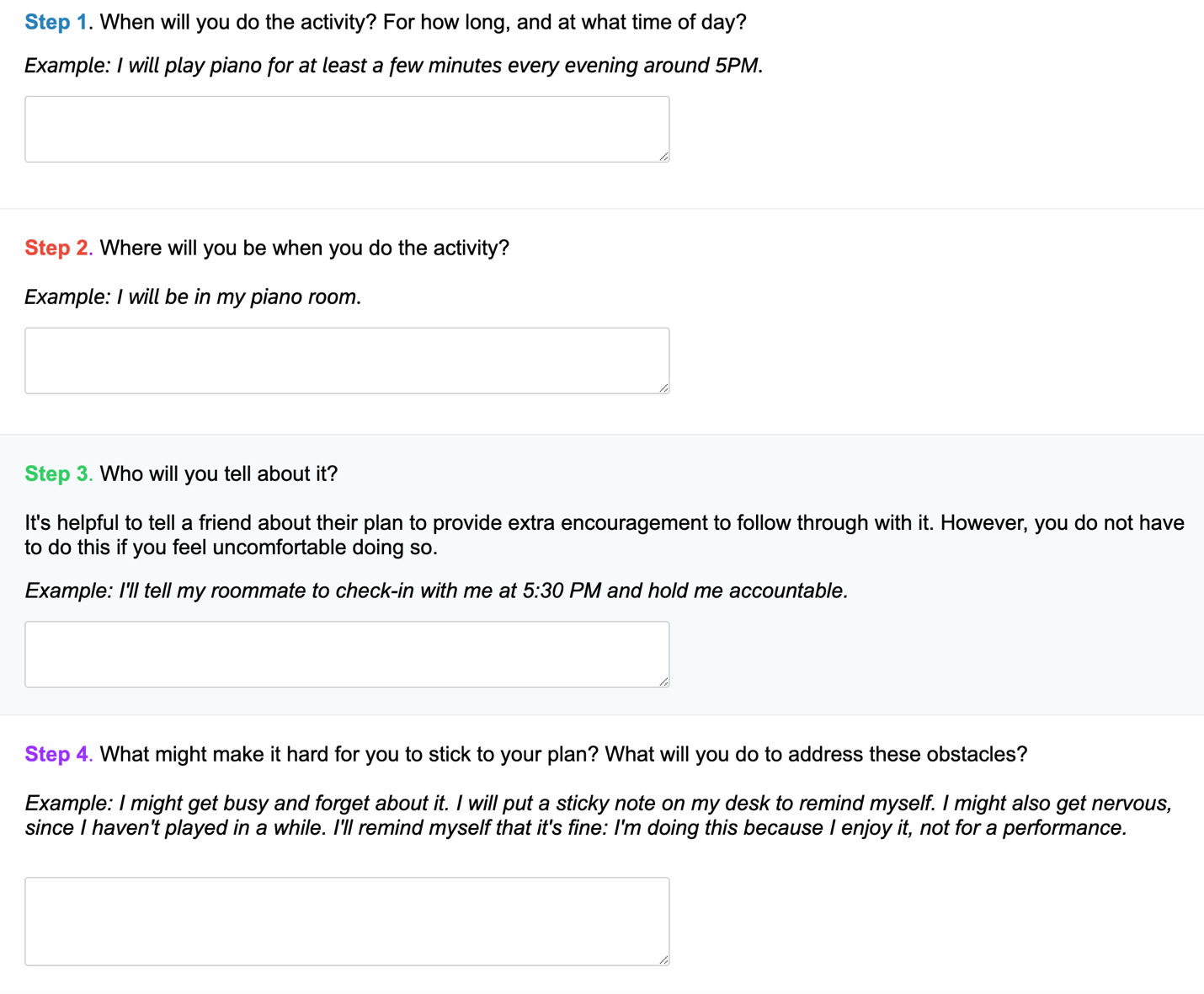


Figure S2

*Gwen’s Story: Vignette for Flexible Thinking Module*


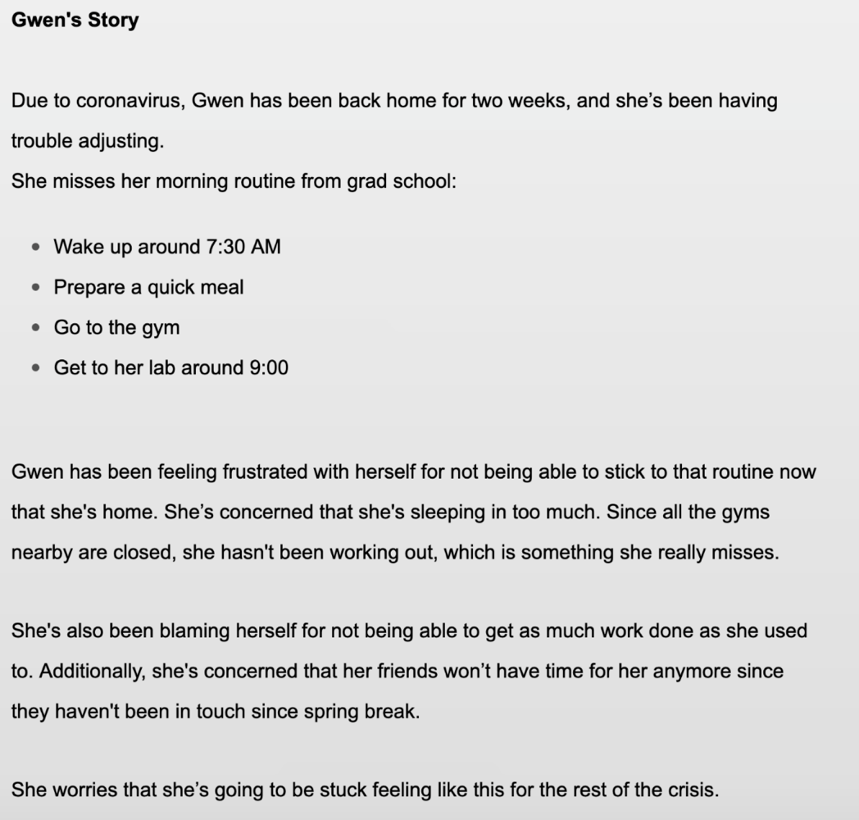


Figure S3

*Reframing Prompt and Example Responses*


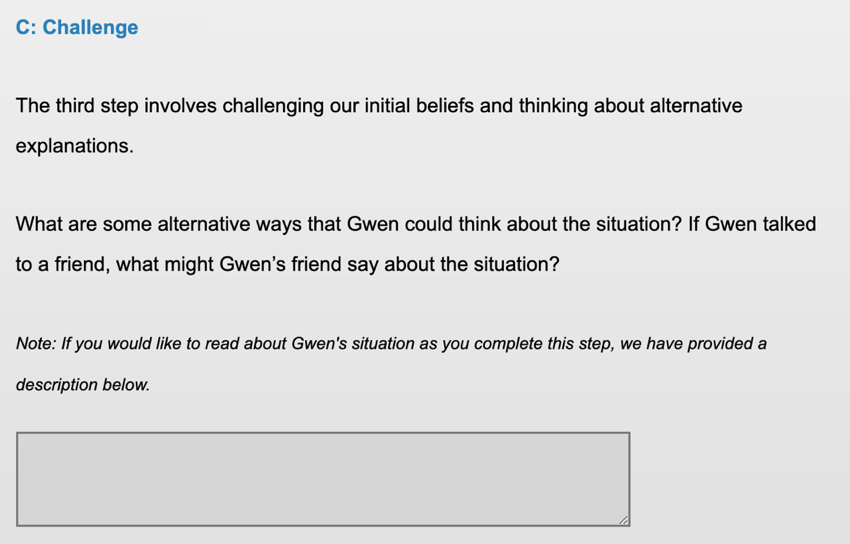

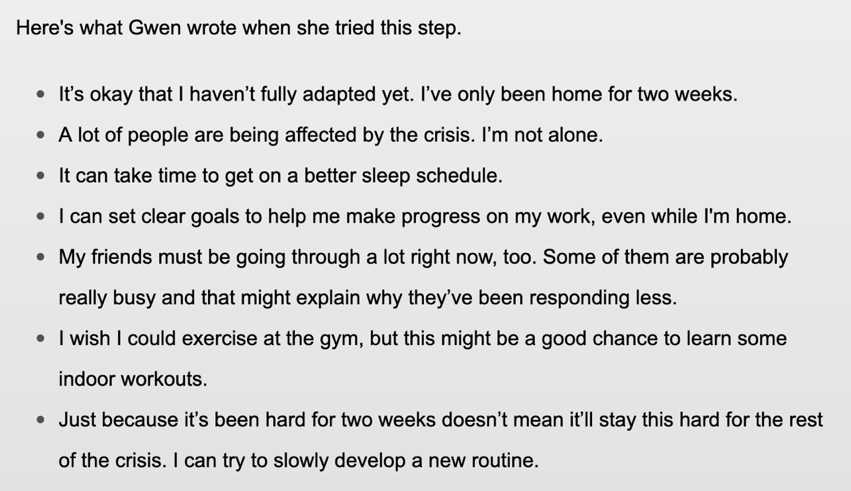


Figure S4

*Three Good Things Prompt and Example Response*


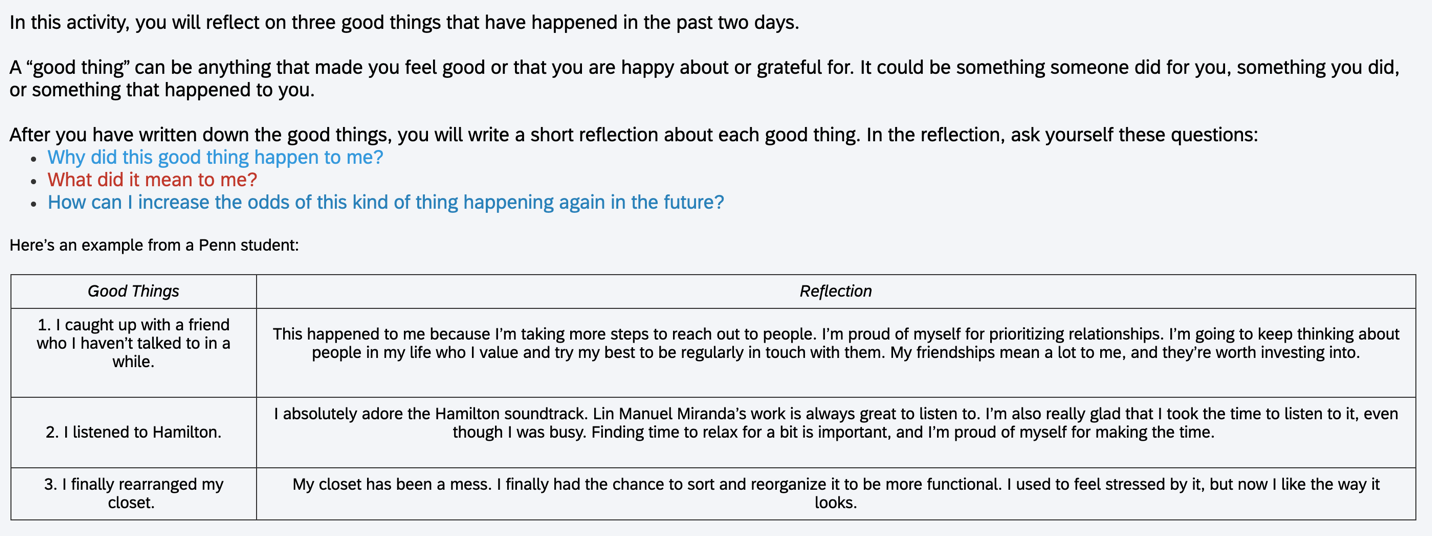


**References**

Chaves, C., Lopez-Gomez, I., Hervas, G., & Vazquez, C. (2017). A comparative study on the

efficacy of a positive psychology intervention and a cognitive behavioral therapy for

clinical depression. *Cognitive Therapy and Research*, *41*, 417-433.

<https://doi.org/10.1002/cpp.2129>

 Chorpita, B. F., & Daleiden, E. L. (2009). Mapping evidence-based treatments for children and adolescents: Application of the distillation and matching model to 615 treatments from 322 randomized trials. *Journal of Consulting and Clinical Psychology*, *77*(3), 566–579. <https://doi.org/10.1037/a0014565>

Dimidjian, S., Hollon, S. D., Dobson, K. S., Schmaling, K. B., Kohlenberg, R. J., Addis, M. E., Gallop, R., McGlinchey, J. B., Markley, D. K., Gollan, J. K., Atkins, D. C., Dunner, D. L., & Jacobson, N. S. (2006). Randomized trial of behavioral activation, cognitive therapy, and antidepressant medication in the acute treatment of adults with major depression. *Journal of Consulting and Clinical Psychology*, *74*(4), 658–670.<https://doi.org/10.1037/0022-006X.74.4.658>

Emmons, R. A., & Stern, R. (2013). Gratitude as a Psychotherapeutic Intervention. *Journal of Clinical Psychology*, *69*(8), 846–855.<https://doi.org/10.1002/jclp.22020>

Higa-McMillan, C. K., Francis, S. E., Rith-Najarian, L., & Chorpita, B. F. (2016). Evidence Base Update: 50 Years of Research on Treatment for Child and Adolescent Anxiety. *Journal of Clinical Child & Adolescent Psychology*, *45*(2), 91–113.<https://doi.org/10.1080/15374416.2015.1046177>

Kushlev, K., Heintzelman, S. J., Lutes, L. D., Wirtz, D., Oishi, S., & Diener, E. (2017). ENHANCE: Design and rationale of a randomized controlled trial for promoting enduring happiness & well-being. *Contemporary Clinical Trials*, *52*, 62–74.<https://doi.org/10.1016/j.cct.2016.11.003>

Murray, L. K., Dorsey, S., Haroz, E., Lee, C., Alsiary, M. M., Haydary, A., Weiss, W. M., & Bolton, P. (2014). A Common Elements Treatment Approach for Adult Mental Health Problems in Low- and Middle-Income Countries. *Cognitive and Behavioral Practice*, *21*(2), 111–123.<https://doi.org/10.1016/j.cbpra.2013.06.005>

Nathan, P. E., & Gorman, J. M. (2015). *A Guide to Treatments That Work*. Oxford University Press.

Patel, V., Weobong, B., Weiss, H. A., Anand, A., Bhat, B., Katti, B., Dimidjian, S., Araya, R., Hollon, S. D., King, M., Vijayakumar, L., Park, A.-L., McDaid, D., Wilson, T., Velleman, R., Kirkwood, B. R., & Fairburn, C. G. (2017). The Healthy Activity Program (HAP), a lay counsellor-delivered brief psychological treatment for severe depression, in primary care in India: A randomised controlled trial. *The Lancet*, *389*(10065), 176–185.<https://doi.org/10.1016/S0140-6736(16)31589-6>

Rashid, T., & Seligman, M. P. (2018). *Positive Psychotherapy: Clinician Manual*. Oxford University Press.

Schleider, J. L., Dobias, M., Sung, J., Mumper, E., & Mullarkey, M. C. (2020). Acceptability and Utility of an Open-Access, Online Single-Session Intervention Platform for Adolescent Mental Health [Preprint]. PsyArXiv. <https://doi.org/10.31234/osf.io/tdrpc>

Schleider, J. L., Mullarkey, M. C., & Chacko, A. (2020). Harnessing Wise Interventions to Advance the Potency and Reach of Youth Mental Health Services. *Clinical Child and Family Psychology Review*, *23*(1), 70–101.<https://doi.org/10.1007/s10567-019-00301-4>

Schleider, J. L., Mullarkey, M. C., Mumper, E., & Sung, J. Y. (2019). The ABC Project: Action Brings Change. *Open Science Framework*. <https://doi.org/10.17605/OSF.IO/QJ94C>

Schleider, J. L., & Weisz, J. R. (2017). Little Treatments, Promising Effects? Meta-Analysis of Single-Session Interventions for Youth Psychiatric Problems. *Journal of the American Academy of Child and Adolescent Psychiatry*, *56*(2), 107–115.<https://doi.org/10.1016/j.jaac.2016.11.007>

Schleider, J. L., & Weisz, J. (2018). A single-session growth mindset intervention for adolescent anxiety and depression: 9-month outcomes of a randomized trial. *Journal of Child Psychology and Psychiatry*, *59*(2), 160–170.<https://doi.org/10.1111/jcpp.12811>

Schueller, S. M. (2010). Preferences for positive psychology exercises. *The Journal of Positive Psychology*, *5*(3), 192–203.<https://doi.org/10.1080/17439761003790948>

Seligman, M. E. P., Rashid, T., & Parks, A. C. (2006). Positive psychotherapy. *The American Psychologist*, *61*(8), 774–788.<https://doi.org/10.1037/0003-066X.61.8.774>

Seligman, M. E. P., Steen, T. A., Park, N., & Peterson, C. (2005). Positive Psychology Progress: Empirical Validation of Interventions. *American Psychologist*, *60*(5), 410–421.<https://doi.org/10.1037/0003-066X.60.5.410>

Taylor, C. T., Lyubomirsky, S., & Stein, M. B. (2017). Upregulating the positive affect system in anxiety and depression: Outcomes of a positive activity intervention: Taylor et al. *Depression and Anxiety*, *34*(3), 267–280.<https://doi.org/10.1002/da.22593>

Walton, G. M. (2014). The New Science of Wise Psychological Interventions. *Current Directions in Psychological Science*, *23*(1), 73–82.<https://doi.org/10.1177/0963721413512856>

Walton, G. M., & Wilson, T. D. (2018). Wise interventions: Psychological remedies for social and personal problems. *Psychological Review*, *125*(5), 617.<https://doi.org/10.1037/rev0000115>

Wasil, A. R., Park, S. J., Gillespie, S., Shingleton, R., Shinde, S., Natu, S., Weisz, J. R., Hollon, S. D., & DeRubeis, R. J. (2020). Harnessing single-session interventions to improve adolescent mental health and well-being in India: Development, adaptation, and pilot testing of online single-session interventions in Indian secondary schools. *Asian Journal of Psychiatry*, *50*, 101980.<https://doi.org/10.1016/j.ajp.2020.101980>

Wasil, A. R., Venturo-Conerly, K. E., Shingleton, R. M., & Weisz, J. R. (2019). A review of popular smartphone apps for depression and anxiety: Assessing the inclusion of evidence-based content. *Behaviour Research and Therapy*, *123*, 103498.<https://doi.org/10.1016/j.brat.2019.103498>

Weisz, J. R., Chorpita, B. F., Palinkas, L. A., Schoenwald, S. K., Miranda, J., Bearman, S. K., Daleiden, E. L., Ugueto, A. M., Ho, A., Martin, J., Gray, J., Alleyne, A., Langer, D. A., Southam-Gerow, M. A., Gibbons, R. D., & Research Network on Youth Mental Health, and the. (2012). Testing Standard and Modular Designs for Psychotherapy Treating Depression, Anxiety, and Conduct Problems in Youth: A Randomized Effectiveness Trial. *Archives of General Psychiatry*, *69*(3), 274–282.<https://doi.org/10.1001/archgenpsychiatry.2011.147>
