# Supplementary material for: Promoting Graduate Student Mental Health During COVID-19: Acceptability, Feasibility, and Perceived Utility of an Online Single-Session Intervention
Source: Front Psychol. 2021 Apr 7;12:569785. doi: 10.3389/fpsyg.2021.569785 (PMC8058455; doi:10.3389/fpsyg.2021.569785)
Supplement: Supplementary file 2 [file Table_2.DOCX]

**Supplement 2: Selected Items from the Secondary Control Scale for Children**

(Weisz, Francis, & Bearman, 2010)

1. When something bad happens, I can find a way to think about it that makes me feel better.
2. After a really hard day, I can make myself feel better by remembering some good things that happened.
3. When bad things happen to me that I can’t control, there are lots of things I can do to feel better.

*Each item is scored on a 4-point scale with the following options: “Very false” (0), “Sort of false” (1), “Sort of true” (2), and “Very true” (3).
