# Supplementary material for: Promoting Graduate Student Mental Health During COVID-19: Acceptability, Feasibility, and Perceived Utility of an Online Single-Session Intervention
Source: Front Psychol. 2021 Apr 7;12:569785. doi: 10.3389/fpsyg.2021.569785 (PMC8058455; doi:10.3389/fpsyg.2021.569785)
Supplement: Supplementary file 3 [file Table_3.docx]

This file includes the R script used for analyses in the manuscript. For questions about the file please contact wasil@sas.upenn.edu

##5.16.20

##Akash R. Wasil and Rose E. Franzen

##In this file, we present our analyses in the order that they appear in the manuscript.

##These analyses were performed in R.

##Please email wasil@sas.upenn.edu with any questions.

#Notes about variable notation:

##Variables that end in ".1" refer to ratings of the first module that participants received (e.g., "AIM.Avg.1" refers to a participant's AIM rating on the first module they received)

##Variables that end in ".2" refer to ratings of the second module that participants received (e.g., "AIM.Avg.2" refers to a participant's AIM rating on the second module they received)

##Variables that end in "T1" refer to pre-intervention measures (e.g., "SCS.Total.T1" refers to a participant's secondary control scale rating at baseline)

##Variables that end in "T2" refer to post-intervention measures (e.g., "SCS.Total.T2" refers to a participant's secondary control scale rating post-intervention).

##Loading Dataset + Renaming/Recoding variables

Data<- read.csv("COMET COVID.csv")

Data<- Data %>%

rename(Mins = ?..Mins)

Data$Age<- as.numeric(Data$Age)

#Method Section: Calculating Cronbach's alphas

PHQMat<- cbind(Data$PHQ1, Data$PHQ2) ##Creating a matrix with the two PHQ items

GADMat<- cbind(Data$GAD1, Data$GAD2) ##Creating a matrix with the two GAD items

SCSMat<- cbind(Data$SCS1, Data$SCS2, Data$SCS3) ##Creating a matrix with the three secondary control items

psych::alpha(PHQMat) ##0.8

psych::alpha(GADMat) ##0.87

psych::alpha(SCSMat) ##0.74

##Results Section: Sample Characteristics and Usage Patterns

## Subsetting to just those who reached first screen of module 1

Starters <-Data[Data$Assigned==1,]

plyr::count(Starters$Completed) ##74 non-completers, 189 completers

189/(74+189) ## 72% completion rate

#Creating two additional subsets: those who finished both modules and those who finished 1 or 0 modules

Completers <-Starters[Starters$Completed==1,]

Noncompleters <-Starters[Starters$Completed==0,]

#Time spent on survey

hist(Completers$Mins)

summary(Completers$Mins) ## Median: 39.23, 1st Q: 26.67

##Table 1: Demographics Table

#Age

describe(Completers$Age) # Mean = 31.04, SD = 8.91

#Sex

plyr::count(Completers$Sex)

127/(127+42+3+2) ##72.99% female

42/(127+42+3+2) ##24.13% male

2/(127+42+3+2)##1.15% Prefer not to answer

3/(127+42+3+2) ## 1.72% Other

# 15 missing

#Race

plyr::count(Completers$Race)

(104+3+5+2)/(37+1+3+9+1+6+5+1+2+2+104) ##66.67% any White

(37+1+3)/(37+1+3+9+1+6+5+1+2+2+104) ## 23.98% any Asian

(1+9+1)/(37+1+3+9+1+6+5+1+2+2+104) ## 6.43% any Black

(1+6+5)/(37+1+3+9+1+6+5+1+2+2+104) ## 7.02% any Hispanic

(1+2)/(37+1+3+9+1+6+5+1+2+2+104) ## 1.75% any Middle Eastern/North African

(2)/(37+1+3+9+1+6+5+1+2+2+104) ## 1.17% any other

#18 missing

#Sexual Orientation

plyr::count(Completers$Sexuality)

(1+1+1+1)/(172) ## 2.33% Asexual

(9+1+2+2+1+1)/172 ## 9.30% Bisexual

(1+1+1)/172 ## 1.74% Demisexual

(1+2+1+1+1)/172 ## 3.49% Fluid

(2+2+1)/172 ## 2.91% Gay or Lesbian

(136+1+1+1+1)/172 ## 81.40% Heterosexual/Straight

(2+1+1+1)/172 ## 2.91% Pansexual

(1+2+2+2+1+2)/172 ## 5.81% Queer

(1+1+1)/172 ## 1.74% Questioning

5/172 ## 2.91% Prefer not to answer

#17 missing

#Social Class

plyr::count(Completers$Income)

30/173*100 ## 17.34% Affluent

111/173*100 ## 64.16% Middle class

5/173*100 ## Poor 2.89%

27/173*100 ## Working class 15.61%

#16 missing

#Personal Experience with Mental Illness

plyr::count(Completers$MH.History)

79/173 #45.67% No

22/173 #12.72% Unsure

72/173 #41.62% Yes

#16 missing

#PHQ, GAD, and SCS by completion status

#PHQ

describe(Completers$PHQ.Total) # Mean 1.98, SD 1.65

describe(Noncompleters$PHQ.Total) # Mean 2.07, SD 1.87

t.test(Completers$PHQ.Total, Noncompleters$PHQ.Total) #ns t=-0.3397, df=115.55, p = 0.7347

cohen.d(Completers$PHQ.Total, Noncompleters$PHQ.Total, na.rm = TRUE)# -0.05

plyr::count(Completers$PHQ.Total)

(21+15+13+7)/(41+39+53+21+15+13+7) # 29.63% would screen positive for likely clinical depression

plyr::count(Noncompleters$PHQ.Total)

(10+6+5+5)/(19+14+13+10+6+5+5) # 36.11% would screen positive for likely clinical depression

#GAD

describe(Completers$GAD.Total) # Mean 2.61, SD 1.86

describe(Noncompleters$GAD.Total) # Mean 2.92, SD 2.03

t.test(Completers$GAD.Total, Noncompleters$GAD.Total) #ns t=-1.1195, df=119.12, p = 0.2652

cohen.d(Completers$GAD.Total, Noncompleters$GAD.Total, na.rm = TRUE) #-0.16

plyr::count(Completers$GAD.Total)

(25+15+15+24)/(19+43+48+25+15+15+24) # 41.80% would screen positive for likely clinical anxiety

plyr::count(Noncompleters$GAD.Total)

(6+7+9+12)/(9+11+18+6+7+9+12) #47.22% would screen positive for likely clinical anxiety

#SCS

describe(Completers$SCS.Total.T1) # Mean 6.01, SD 1.82

describe(Noncompleters$SCS.Total.T1) # Mean 6.25, SD 1.68

t.test(Completers$SCS.Total.T1, Noncompleters$SCS.Total.T1) #ns t=-0.97669, df=130.8

cohen.d(Completers$SCS.Total.T1, Noncompleters$SCS.Total.T1, na.rm = TRUE) #-0.13

# Acceptability and Perceived Utility

##Completers, Applicability/AIM:

##Completers AIM items

###Creating overall average scores on AIM for completers

####For those who did not fill out AIM after completing second module, their "average score" is equivalent to their score on the first module. For those who provided AIM ratings after both modules, their average score is the average of the two.

Completers$AvgApproval<-ifelse(is.na(Completers$Approval.2),Completers$Approval.1,(Completers$Approval.1+Completers$Approval.2)/2)

Completers$AvgLike<-ifelse(is.na(Completers$Like.2),Completers$Like.1,(Completers$Like.1+Completers$Like.2)/2)

Completers$AvgWelcome<-ifelse(is.na(Completers$Welcome.2),Completers$Welcome.1,(Completers$Welcome.1+Completers$Welcome.2)/2)

Completers$AvgAppeal<-ifelse(is.na(Completers$Appeal.2),Completers$Appeal.1,(Completers$Appeal.1+Completers$Appeal.2)/2)

Completers$AvgAIM<-((Completers$AvgAppeal + Completers$AvgApproval + Completers$AvgLike + Completers$AvgWelcome)/4)

describe(Completers$AvgApproval) ## mean=4.18, SD=0.68

describe(Completers$AvgLike) # 4.14, 0.69

describe(Completers$AvgWelcome) #4.23, 0.69

describe(Completers$AvgAppeal) #4.12, 0.70

describe(Completers$AvgAIM) #4.17, 0.65

#Noncompleters AIM items

##Creating "Averages" in order to keep column titles parallel between Completers and noncompleters to allow later use of rbind

Noncompleters$AvgApproval<- Noncompleters$Approval.1

Noncompleters$AvgLike<- Noncompleters$Like.1

Noncompleters$AvgWelcome<- Noncompleters$Welcome.1

Noncompleters$AvgAppeal<- Noncompleters$Appeal.1

Noncompleters$AvgAIM<- ((Noncompleters$AvgAppeal + Noncompleters$AvgApproval + Noncompleters$AvgLike + Noncompleters$AvgWelcome)/4)

describe(Noncompleters$AvgApproval) #4.0, 0.82

describe(Noncompleters$AvgLike) #3.88, 0.9

describe(Noncompleters$AvgWelcome) #3.88, 0.9

describe(Noncompleters$AvgAppeal) #4.0, 0.76

describe(Noncompleters$AvgAIM)#3.96, 0.79

# Comparing acceptability rating of first module between completers and non-completers

t.test(Completers$AIM.Avg.1, Noncompleters$AIM.Avg.1) #ns t=1.0885, df=28.861

cohen.d(Completers$AIM.Avg.1, Noncompleters$AIM.Avg.1, na.rm = TRUE) #0.24

describe(Completers$AIM.Avg.1) #4.14, 0.76

#Comparing acceptability rating of first module against second module for completers

t.test(Completers$AIM.Avg.1, Completers$AIM.Avg.2, paired = TRUE) #ns t = -0.8372, df=179

cohen.d(Completers$AIM.Avg.1, Completers$AIM.Avg.2, na.rm=TRUE)#-0.06

describe(Completers$AIM.Avg.2) ## mean = 4.19, SD=0.77

#Percentage of completers with acceptability ratings>3

Completers$AIMThreshold<- ifelse(Completers$AvgAIM>3, 1, 0)

plyr::count(Completers$AIMThreshold) ##175 above three, 10 below or equal to three, 4 NA

175/185 ## 95% of completers provided a rating >3

#Percentage of non-completers with acceptability ratings>3

Noncompleters$AIMThreshold<-ifelse(Noncompleters$AIM.Avg.1>3, 1, 0)

plyr::count(Noncompleters$AIMThreshold) ##20 above three, 4 below or equal to three, 50 NA (high number of NA due to many noncompleters finishing 0 modules)

20/24 ##83.3% of non-completers provided a rating >3

(175+20)/(185+24) ## 93% of participants provided a rating >3

##Chi-square to compare acceptability endorsement between completers and non-completers

CompletersAIM<- c(175, 10)

NonCompletersAIM<- c(20, 4)

AIMMat<- cbind(CompletersAIM, NonCompletersAIM)

chisq.test(AIMMat, correct=FALSE) #4.3106, p = 0.03737

##Differences in Acceptability

Starters<-rbind(Completers, Noncompleters)

anova(lm(AvgAIM~Sex,data = Starters)) ##ns

anova(lm(AvgAIM~Age,data = Starters)) ##ns

anova(lm(AvgAIM~PHQ.Total,data = Starters)) ##ns

anova(lm(AvgAIM~GAD.Total,data = Starters)) ##ns

anova(lm(AvgAIM~Income,data = Starters)) ##ns

anova(lm(AvgAIM~SCS.Total.T1,data = Starters)) ##p = 0.002034

#investigating relationship between secondary control and AIM

AIMControl<- lm(AvgAIM~SCS.Total.T1,data = Starters)

summary(AIMControl)

cor.test(Starters$AvgAIM, Starters$SCS.Total.T1) #r=0.21, p = 0.002

##Investigating AIM scores at each level of secondary control

median(Completers$AvgAIM[Data$SCS.Total.T1==1], na.rm = TRUE)

median(Completers$AvgAIM[Data$SCS.Total.T1==2], na.rm = TRUE)

median(Completers$AvgAIM[Data$SCS.Total.T1==3], na.rm = TRUE)

median(Completers$AvgAIM[Data$SCS.Total.T1==4], na.rm = TRUE)

median(Completers$AvgAIM[Data$SCS.Total.T1==5], na.rm = TRUE)

median(Completers$AvgAIM[Data$SCS.Total.T1==6], na.rm = TRUE)

median(Completers$AvgAIM[Data$SCS.Total.T1==7], na.rm = TRUE)

median(Completers$AvgAIM[Data$SCS.Total.T1==8], na.rm = TRUE)

median(Completers$AvgAIM[Data$SCS.Total.T1==9], na.rm = TRUE) # median score is above 3 at each level

# Perceived Utility

#Completers Utility Items

###Creating overall average scores on Perceived Utility for completers using same logic as used above for AIM.

Completers$AvgHelpful<-ifelse(is.na(Completers$Help.2),Completers$Help.1,(Completers$Help.1+Completers$Help.2)/2)

Completers$AvgEngaging<-ifelse(is.na(Completers$Engage.2),Completers$Engage.1,(Completers$Engage.1+Completers$Engage.2)/2)

Completers$AvgApplicable<-ifelse(is.na(Completers$Apply.2),Completers$Apply.1,(Completers$Apply.1+Completers$Apply.2)/2)

Completers$AvgUtility<- ((Completers$AvgHelpful+Completers$AvgEngaging+Completers$AvgApplicable)/3)

describe(Completers$AvgHelpful) #5.73, 1.01

describe(Completers$AvgEngaging) #5.57, 1.07

describe(Completers$AvgApplicable) #5.67, 0.97

describe(Completers$AvgUtility)#5.66, 0.93

#Noncompleters Perceived Utility Items

##Creating "Averages" in order to keep column titles parallel between Completers and noncompleters to allow later use of rbind

Noncompleters$AvgHelpful<- Noncompleters$Help.1

Noncompleters$AvgEngaging<- Noncompleters$Engage.1

Noncompleters$AvgApplicable<- Noncompleters$Apply.1

Noncompleters$AvgUtility<- (Noncompleters$AvgHelpful+Noncompleters$AvgEngaging+Noncompleters$AvgApplicable)/3

##Creating a new column; this will allow us to use rbind later on

Noncompleters$AvgUtility.1<-(Noncompleters$AvgUtility)

describe(Noncompleters$Help.1) # 5.52, 1.19

describe(Noncompleters$Engage.1) #5.48, 1.08

describe(Noncompleters$Apply.1) #5.48, 1.08

describe(Noncompleters$AvgUtility.1) #5.49, 1.06

#Percentage who provided perceived utility ratings >4

Completers$HelpThreshold<- ifelse(Completers$AvgHelpful>4, 1, 0)

plyr::count(Completers$HelpThreshold) ##166 above four, 19 equal to or below four, 4 na

166/185 # 90% of completers provided a rating >4

Noncompleters$HelpThreshold<- ifelse(Noncompleters$AvgHelpful>4, 1, 0)

plyr::count(Noncompleters$HelpThreshold) #19 above four, 6 equal to or below four, 49 na (high number of NA due to many noncompleters finishing 0 modules)

19/25 # 76% of noncompleters provided a rating >4

(166+19)/(185+25) ## 88% of all participants provided a rating >4

Completers$EngageThreshold<- ifelse(Completers$AvgEngaging>4, 1, 0)

plyr::count(Completers$EngageThreshold) ##160 above four, 25 equal to or below four, 4 na

160/185 # 86% of completers provided a rating >4

Noncompleters$EngageThreshold<- ifelse(Noncompleters$AvgEngaging>4, 1, 0)

plyr::count(Noncompleters$EngageThreshold) ##21 above four, four below or equal to four, 49 na (high number of NA due to many noncompleters finishing 0 modules)

21/25 # 84% of noncompleters provided a rating >4

(160+21)/(25+185) # 86% of participants provided a rating >4

Completers$ApplyThreshold<- ifelse(Completers$AvgApplicable>4, 1, 0)

plyr::count(Completers$ApplyThreshold) ##164 above four, 21 below or equal to four, 4 na

164/185 # 89% of completers provided a rating >4

Noncompleters$ApplyThreshold<- ifelse(Noncompleters$AvgApplicable>4, 1, 0)

plyr::count(Noncompleters$ApplyThreshold) ##19 above four, 6 below or equal to four, 49 na (high number of NA due to many noncompleters finishing 0 modules)

19/25 # 76% of noncompleters provided a rating >4

(164+19)/(185+25) # 87% of participants provided a rating

Completers$UtilityThreshold<- ifelse(Completers$AvgUtility>4, 1, 0)

plyr::count(Completers$UtilityThreshold) ##170 above four, 15 below or equal to four, 4 na

170/185 # 92% of noncompleters provided a rating >4

Noncompleters$UtilityThreshold<- ifelse(Noncompleters$AvgUtility>4, 1, 0)

plyr::count(Noncompleters$UtilityThreshold) ##21 above four, 4 below or equal to four, 49 na (high number of NA due to many noncompleters finishing 0 modules)

21/25 # 84% of noncompleters provided a rating >4

CompletersUtility<- c(170, 15)

NonCompletersUtility<- c(21, 4)

UtilityMat<- cbind(CompletersUtility,NonCompletersUtility)

chisq.test(UtilityMat, correct = FALSE) # 1.6669, p=0.1967

# Comparing utility rating of first module between completers and non-completers

Completers$AvgUtility.1<-((Completers$Help.1+Completers$Engage.1+Completers$Apply.1)/3)

describe(Completers$AvgUtility.1) #5.61, 1

describe(Noncompleters$AvgUtility.1) #5.49, 1.06

t.test(Completers$AvgUtility.1, Noncompleters$AvgUtility)# t=0.52989, df=30.066 #p = 0.6001

cohen.d(Completers$AvgUtility.1, Noncompleters$AvgUtility, na.rm=TRUE) #0.118

#Comparing utility rating of first module against second module for completers

Completers$AvgUtility.2<- ((Completers$Help.2+Completers$Engage.2+Completers$Apply.2)/3)

t.test(Completers$AvgUtility.1, Completers$AvgUtility.2, paired = TRUE) # t = -1.1337, df=180, p = 0.2584

cohen.d(Completers$AvgUtility.1, Completers$AvgUtility.2, na.rm=TRUE)#-0.09

describe(Completers$AvgUtility.2) ## mean = 5.71, SD=1.15

##Differences in Utility

#Creating Noncompleters utility 2 just to allow for rbind

Noncompleters$AvgUtility.2<- NA

Starters<-rbind(Completers, Noncompleters)

UtilityCorMat<- cbind(Starters$AvgHelpful, Starters$AvgEngaging, Starters$AvgApplicable)

UtilityCorMat<-as.data.frame(UtilityCorMat)

cor(UtilityCorMat, use = "complete.obs")

cor(UtilityCorMat, use = "pairwise.complete.obs")

anova(lm(AvgUtility~Sex,data = Starters)) ##ns

anova(lm(AvgUtility~Age,data = Starters)) ##ns

anova(lm(AvgUtility~PHQ.Total,data = Starters)) ##ns

anova(lm(AvgUtility~GAD.Total,data = Starters)) ##ns

anova(lm(AvgUtility~Income,data = Starters)) ##ns

anova(lm(AvgUtility~SCS.Total.T1,data = Starters)) ## p = 0.001763

#Investigating relationship between perceived utility and secondary control

UtilityControl<- lm(AvgUtility~SCS.Total.T1,data = Starters)

summary(UtilityControl)

cor.test(Starters$AvgUtility, Starters$SCS.Total.T1) #r=0.21, p = 0.002

##Investigating perceived utility at each level of secondary control

median(Completers$AvgUtility[Data$SCS.Total.T1==1], na.rm = TRUE)

median(Completers$AvgUtility[Data$SCS.Total.T1==2], na.rm = TRUE)

median(Completers$AvgUtility[Data$SCS.Total.T1==3], na.rm = TRUE)

median(Completers$AvgUtility[Data$SCS.Total.T1==4], na.rm = TRUE)

median(Completers$AvgUtility[Data$SCS.Total.T1==5], na.rm = TRUE)

median(Completers$AvgUtility[Data$SCS.Total.T1==6], na.rm = TRUE)

median(Completers$AvgUtility[Data$SCS.Total.T1==7], na.rm = TRUE)

median(Completers$AvgUtility[Data$SCS.Total.T1==8], na.rm = TRUE)

median(Completers$AvgUtility[Data$SCS.Total.T1==9], na.rm = TRUE) # median score is above 4 at each level

##Pre-Post Analyses

##Secondary Control

t.test(Completers$SCS.Total.T1, Completers$SCS.Total.T2, paired = TRUE)# t=-6.5337, df=173, p=0.0000000006909

describe(Completers$SCS.Total.T1) ##mean=6.01, SD=1.82

describe(Completers$SCS.Total.T2) ##mean=6.64, SD=1.64

cor.test(Completers$SCS.Total.T1, Completers$SCS.Total.T2) #r = 0.738 #calculated for use in Lakens cohen's d calculator

##Impact of COVID-19 on Quality of Life

t.test(Completers$COVID.Impact.T1, Completers$COVID.Impact.T2, paired = TRUE) # t=3.5074, df=178, p = 0.0005728

describe(Completers$COVID.Impact.T1) ##mean=3.94, SD=1.61

describe(Completers$COVID.Impact.T2) ##mean=3.6, SD=1.53

cor.test(Completers$COVID.Impact.T1, Completers$COVID.Impact.T2) #r = 0.630 #calculated for use in Lakens cohen's d calculator

##Ability to Handle COVID-related Changes

t.test(Completers$Handle.COVID.Changes.T1, Completers$Handle.COVID.Changes.T2, paired = TRUE) ##ns; t=-1.8504 df= 178 p=0.066

describe(Completers$Handle.COVID.Changes.T1) ##mean=5.5, SD= 1.25

describe(Completers$Handle.COVID.Changes.T2) ##mean=5.64, SD= 1.05

cor.test(Completers$Handle.COVID.Changes.T1, Completers$Handle.COVID.Changes.T2)# r = 0.627 #calculated for use in Lakens cohen's d calculator

#Program Could Help Handle Coronavirus Challenges

describe(Completers$Handle.Challenges) ##mean= 5.67, SD = 1.17

Completers$HandleThreshold<- ifelse(Completers$Handle.Challenges>4, 1, 0)

plyr::count(Completers$HandleThreshold) ##159 above four, 21 below or equal to four, 9 NA

159/180 # 88% completers endorsed

##Pre-post effect Sizes were calculated using the calculator provided by Lakens (2013). See https://osf.io/ixgcd/

##See also:

##Lakens, D. (2013). Calculating and reporting effect sizes to facilitate cumulative science: A practical primer for t-tests and ANOVAs. Frontiers in Psychology, 4. https://doi.org/10.3389/fpsyg.2013.00863
